# Time-restricted eating versus dietetic guidance on glycaemic outcomes in adults at risk of type 2 diabetes: a non-inferiority randomised clinical trial

**DOI:** 10.1007/s00125-026-06762-x

**Published:** 2026-06-06

**Authors:** Evelyn B. Parr, Rasha Charrouf, Amy T. Hutchison, Steve A. Flint, Xiao Tong Teong, Andrew D. Vincent, Ana Paula Bravo-Garcia, Zoe Siviour, Gary A. Wittert, Leah Brennan, Brooke L. Devlin, John A. Hawley, Leonie K. Heilbronn

**Affiliations:** 1 Centre for Human Metabolism and Performance, Mary MacKillop Institute for Health Research, Australian Catholic University, Melbourne, VIC Australia; 2 School of Medicine, Adelaide University, Adelaide, SA Australia; 3 Lifelong Health Theme, South Australian Health and Medical Research Institute, Adelaide, SA Australia; 4 Diabetes Technology Research Group, Department of Medicine, The University of Melbourne, Melbourne, VIC Australia; 5 School of Psychology and Public Health, La Trobe University, Wodonga, VIC Australia; 6 School of Human Movement and Nutrition Sciences, The University of Queensland, Brisbane, QLD Australia; 7 Department of Sport and Exercise Sciences, Manchester Metropolitan University Institute of Sport, Manchester, UK

**Keywords:** Dietary counselling, HbA_1c_, Intermittent fasting, Lifestyle intervention, Non-inferiority, Obesity, Telemedicine, Weight loss

## Abstract

**Aims/hypothesis:**

Time-restricted eating (TRE) consolidates daily energy intake to a consistent 6–10 h window. The aim was to assess whether TRE is non-inferior to individualised dietetic guidance (IDG) in changing HbA_1c_ at 4 months in adults at risk of type 2 diabetes.

**Methods:**

In a two-arm, parallel-group, multi-centre randomised, non-inferiority clinical trial at two Australian clinical research institutes, adults at risk of type 2 diabetes (i.e. with overweight or obesity and a score of ≥15 on the Australian type 2 diabetes risk assessment tool) were recruited. Participants were randomly assigned using an electronic system (1:1) to TRE (9 h, self-selected eating window, last eating occasion by 19:00 hours) or IDG. Staff conducting assessments and analyses were blinded to allocation; participants and dietitians were unblinded. Both groups received five personalised telehealth consultations between 0 and 3 months (3 h total), specific to their allocated group. The primary outcome was the between-group difference for change in HbA_1c_ at 4 months assessed via covariate linear regression using multiple imputation with a non-inferiority margin set at 1.1 mmol/mol (0.10%). Secondary outcomes were change in HbA_1c_ at 12 months and changes in fasting glucose, insulin, HOMA-IR and nocturnal glucose AUC. Exploratory outcomes included changes in cardiometabolic outcomes and body mass and composition. Adverse events were tracked for 12 months.

**Results:**

One hundred and twenty four participants were randomised to TRE and 123 to IDG (mean ± SD HbA_1c_: 40 ± 3 mmol/mol [5.8 ± 0.3%]). At 4 months, TRE was non-inferior but not superior to IDG for HbA_1c_ (mean [95% CI]: −0.22 [−0.72, 0.30] mmol/mol; −0.02 [−0.07, 0.03]%; *p*=0.40). At 12 months, the upper bound of the difference between groups was larger than the non-inferiority margin (0.10%) meaning that non-inferiority could no longer be concluded (mean [95% CI]: 0.47 [−0.19, 1.23] mmol/mol; 0.05 [−0.02, 0.11]%; *p*=0.14). However, the absolute changes in HbA_1c_ were small and not clinically meaningful in either group at either time point. Adverse events were minor and not different between interventions.

**Conclusions/interpretation:**

TRE may offer a pragmatic and practical short-term alternative when access to dietetic support is limited, or if the approach is preferred by an individual.

**Trial registration:**

ClinicalTrials.gov; NCT04762251

**Funding:**

This research was funded by the Australian Government Medical Research Future Fund (MRFF 1200555).

**Graphical Abstract:**

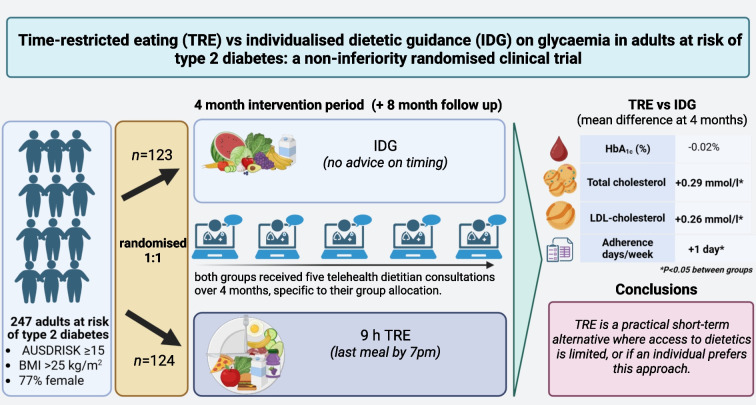

**Supplementary Information:**

The online version of this article (10.1007/s00125-026-06762-x) contains peer-reviewed but unedited supplementary material.


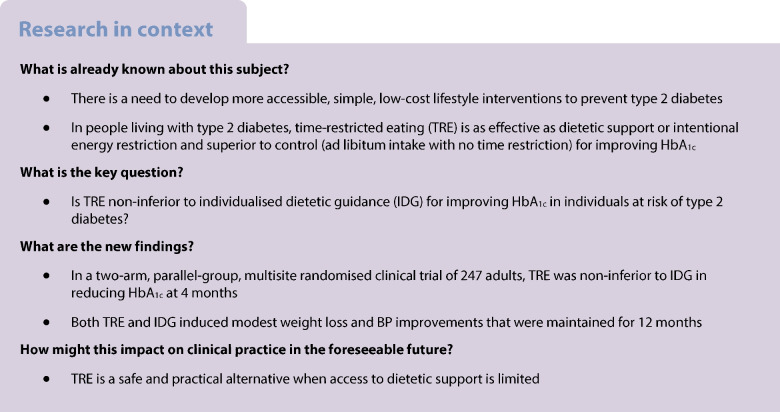


## Introduction

Type 2 diabetes is a progressive and chronic health condition that is estimated to affect 1.27 billion people globally by 2050 [1]. Lifestyle modifications, particularly dietary change and the resulting weight loss are effective in delaying or preventing the development of type 2 diabetes [2, 3]. However, lifestyle modifications can be costly for individuals to implement and difficult to initiate and sustain. Furthermore, dietary support is less accessible for those living in rural and remote areas or for those with socioeconomic disadvantage [4]. There is a need to develop more accessible, simple, low-cost lifestyle interventions to prevent type 2 diabetes.

Time-restricted eating (TRE) is a dietary strategy that simply confines eating occasions to a consistent 6–10 h window, predominately during daytime hours [5]. In clinical trials lasting up to 3 months, TRE was safe and feasible, and induced modest weight loss and cardiometabolic benefit as compared with no diet control in people living with obesity or at risk of type 2 diabetes [6–11]. The benefits of TRE appear to be at least partially underpinned by modest weight loss, which occurs due to an incidental energy restriction induced by a reduced eating window.

Most free-living trials of TRE have set weight loss as the primary outcome, have compared against no-intervention control and are short-term [9, 10, 12–14], or have combined TRE with energy restriction [15, 16], with only one study reporting on TRE alone out to 12 months [17]. Several studies have explored the effects of TRE on glycaemic outcomes, with some trials reporting reductions in fasting glucose [8] and fasting insulin [6, 9, 12], and improved beta cell responsiveness [6]. Two separate meta-analyses of 17 trials with >12 weeks intervention [18] and 27 trials [19] showed an overall modest benefit of TRE for body weight and glycaemia, although there was high heterogeneity among studies, and the certainty of the evidence was low.

In contrast, in people living with type 2 diabetes, TRE was as effective as dietetic support [20] or intentional energy restriction [21] and superior to control (ad libitum intake with no time restriction) [22] for HbA_1c_. Adding TRE to Mediterranean diet advice also produced a 1.1 mmol/mol (0.1%) greater benefit for HbA_1c_ when compared with standard Mediterranean diet advice alone in 108 participants with the metabolic syndrome (including elevated fasting glucose concentration or elevated HbA_1c_) after 3 months [23].

The effectiveness of TRE alone against gold standard dietary approaches in people without diabetes is under-researched. Despite dietetic advice being the first-line approach for reducing the risk of type 2 diabetes, no previous studies have compared TRE against dietetic counselling tailored to an individual at risk of type 2 diabetes. The aim of this randomised, multisite clinical trial was to test whether TRE alone is non-inferior to individualised dietetic guidance (IDG) for glycaemic and cardiometabolic outcomes in adults at risk of developing type 2 diabetes at 4 months, with a further 8 month follow-up. It was hypothesised that TRE would be non-inferior to IDG in reducing HbA_1c_ at 4 months.

## Methods

### Study design

This parallel-group, randomised, multi-centre, non-inferiority clinical trial, conducted between 17 February 2021 and 19 April 2024, involved a 4 month active intervention phase, with an 8 month follow-up period. The study was approved by The Central Adelaide Local Health Network Human Research Ethics Committee and Australian Catholic University Human Research Ethics Committee. All participants provided written informed consent. An independent Data and Safety Monitoring Committee (DSMC) approved the final protocol and provided ongoing oversight for the trial duration. The trial protocol has been published [24], with further details in electronic supplementary material (ESM) 1. This trial report follows the Consolidated Standards of Reporting Trials (CONSORT) reporting guidelines and checklist.

### Study population and randomisation

Two hundred and forty seven eligible participants (aged 35–70 years, BMI 25–45 kg/m^2^, a score of ≥15 on the Australian type 2 diabetes risk assessment tool [AUSDRISK] [25], weight change ≤5% for ≥6 months, without a diagnosis of type 2 diabetes, not taking medications that could affect glucose metabolism or weight management, self-reported eating window ≥12 h/day, and not engaged in night-shift work) were randomised to TRE or IDG in a 1:1 ratio. Complete exclusion and inclusion criteria are detailed in ESM 1. Randomisation was block-stratified by site and baseline HbA_1c_ (<39 mmol/mol [<5.7%]; ≥39 mmol/mol [≥5.7%] to 48 mmol/mol [6.5%]), with random block lengths 2 or 4, performed in REDCap according to a randomisation list generated by the study statistician (ADV).

### Diet interventions

**Fig. 1:**
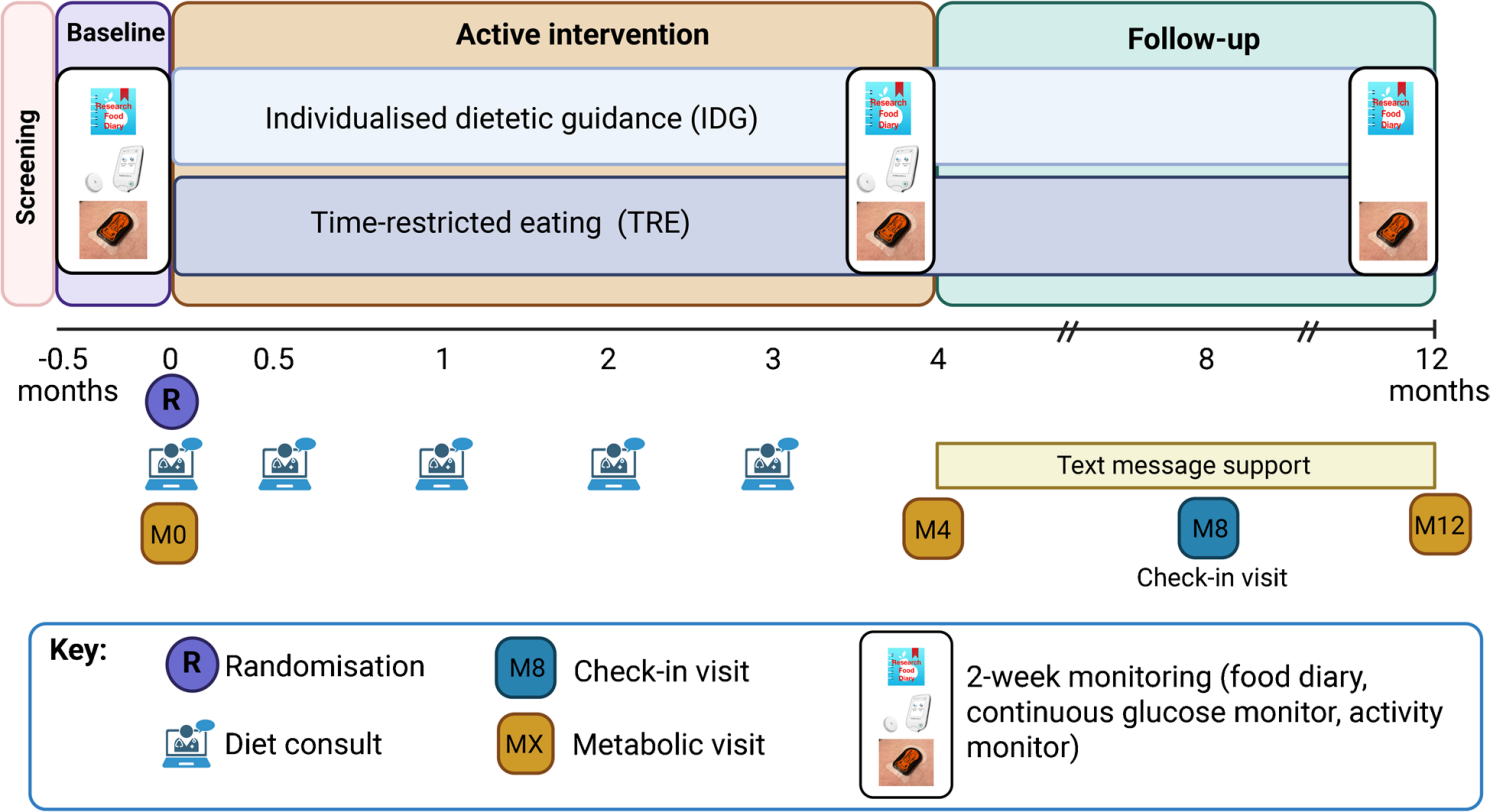
Timeline of study visits and assessments. After initial screening, participants attended an in-person visit where food diaries were explained, continuous glucose monitors and activity monitors were fitted and worn for 2 weeks prior to the month 0 (M0) metabolic visit at which clinical endpoints were collected in the fasting state. Randomisation took place after M0, and five diet consultations were scheduled at M0 (DC1), M0.5 (DC2), M1 (DC3), M2 (DC4) and M3 (DC5). Additional monitoring visits occurred at M3.5 and M11.5 and metabolic visits at M4 and M12. An in-person check-in also occurred at M8. DC, diet consultation; M metabolic visit. Created in BioRender. Hutchison, A. (2026) https://BioRender.com/jrp8dr8

To ensure a consistent approach and time-matched support, both groups received five telehealth consultations with an Accredited Practising Dietitian (APD) over 4 months (Fig. 1). At each session, participants set individual goals, which guided subsequent visits and helped maintain motivation. Advice was customised to align with participants’ cultural and personal preferences.

The TRE group was instructed to limit their daily food intake to a self-selected eating window of 9 h per day (the time between the first and the last energy intake each day) over 12 months, with the last energy intake of the day being before 19:00 hours. The TRE participants were also provided with strategies to promote adherence. These included preparing meals and snacks in advance to reduce unplanned eating outside the eating window, establishing consistent daily routines, planning for social occasions, and strategies to manage hunger during fasting periods (e.g. hydration, non‑energy beverages). Participants were also encouraged to reflect on barriers to adherence and to problem‑solve collaboratively with the dietitian across consultations. Outside the self-selected eating window, TRE participants were permitted to consume water, black coffee and/or herbal tea. Although advice for the TRE group was delivered by an APD, no dietary guidance was provided and the general support provided could have been delivered by a diabetes educator, health coach or nutritionist.

In the IDG group, dietary advice was provided by an APD in line with type 2 diabetes current practice guidelines [26, 27], Australian Dietary Guidelines [28] and standard practice, to improve diet quality and strategies to promote adherence. Specifically, IDG participants were provided with a handout covering suggested foods to enjoy (i.e. healthy/nutritious foods), foods to limit, tips for healthy eating, limiting discretionary foods and appropriate serving size; this was used to provide consistent baseline information at the first consultation. Consultations covered key topics including alcohol, carbohydrate intake, healthy eating, heart health, label reading and weight management. 

Both groups were encouraged to increase their physical activity [24]. At the end of the 4 month active intervention, participants were instructed to continue adhering to their respective intervention until the end of the study (12 months). From month 4, participants received text messages with practical tips to overcome barriers and maintain adherence (see [24]).

### Outcome measures

The primary outcome was the change in HbA_1c_ at 4 months. Secondary efficacy outcomes were changes in HbA_1c_ at 12 months, fasting glucose and fasting insulin concentrations, HOMA-IR at 4 and 12 months, and the change in nocturnal mean glucose (midnight to 04:00 hours, given this period is most amenable to TRE) at 4 months. Exploratory outcomes included changes in body mass, body composition, BP, blood lipids, liver markers, serum high-sensitivity C-reactive protein (hs-CRP), 24 h assessment of glycaemia by continuous glucose monitoring (CGM), sleep duration, physical activity, meal timing and dietary intake. Methods of outcome assessments are detailed within the protocol [24] and self-reported adherence is detailed in ESM 2 (Methods). Body mass and composition were assessed fasted, while the participant wore a gown, using scales and dual-energy x-ray absorptiometry (GE Lunar iDXA Pro, USA), respectively. Estimated visceral adipose tissue (eVAT) was assessed using the GE Lunar software (encore Version 16). Blood samples were collected between 07:00 and 10:00 hours, after fasting (from 19:00 hours) (consistent within participant). Meal timing and dietary intake were objectively obtained from food photos and food diaries (using Research Food Diary [the research version of Easy Diet Diary; Xyris Software Pty, Australia], which provides no energy or macronutrient feedback), respectively, according to previously developed criteria [20] from 5 days (three weekdays and two weekend days) of each of the 2 week measurement periods (baseline, month 4 and month 12). Blinded CGM sensors (FreeStyle Libre Pro iQ; Abbott, Germany) were inserted by a research team member to the upper arm of the participants’ choice, at baseline and 4 months, and worn for up to 14 days (removed at the 4 month visit). Where a CGM sensor dislodged within 3 days, it was replaced where possible. Resulting CGM data were assessed for mean nocturnal glucose concentrations (between midnight and 04:00 hours), time in range (3.9–7.8 mmol/l glucose), time below range (<3.9 mmol/l glucose), time above range (>7.8 mmol/l glucose), 24 h incremental AUC (iAUC) [29] and 24 h SD when at least 24 h of data were available. Physical activity data, including time in bed, were collected using thigh-worn inclinometers (ActivPAL; PAL Technologies, Scotland) for the 14 day monitoring periods and validated using participant self-reported wake and bedtime.

### Safety outcomes

During each metabolic visit, participants used a REDCap questionnaire to report any health-related conditions or physical symptoms (such as fatigue, constipation, headache or light-headedness) since the preceding visit. Adverse events were recorded and categorised according to Common Terminology Criteria for Adverse Events (CTCAE), and all serious adverse events were reported to the DSMC.

### Sample size

**Fig. 2:**
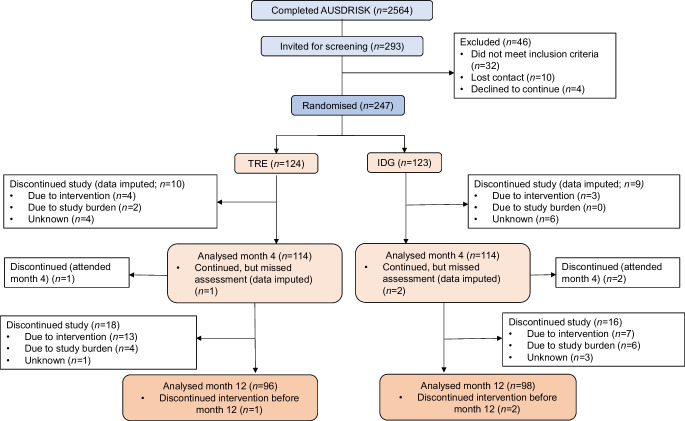
CONSORT study flow diagram. Participants were randomised into either TRE (*n*=124) or IDG (*n*=123) groups. Primary outcome analyses were intention to treat, where all participants, regardless of discontinuation status, were included in the imputed analyses. Individuals who elected to discontinue the study were invited to attend the next assessment visit following their discontinuation

Assuming a within-group SD of ≤0.6% [2, 30], a pre–post HbA_1c_ correlation of 0.65 and attrition rate of ≤10%, then with *n*=214 (randomised 1:1) there is at least 80% power to conclude that TRE is non-inferior to IDG with a margin of 1.1 mmol/mol (0.10%) in a baseline-adjusted ANCOVA when the true difference is 0.10% in favour of TRE (two-sided α=0.05) [31]. Additional details of this calculation are presented in Charrouf et al [24]. Initially, we anticipated an attrition rate of <20% at 4 months, requiring 268 participants. Due to a lower-than-expected attrition rate during the study (Fig. 2), projected attrition was revised to 10% and the recruitment target was reduced accordingly (*n*=247) as approved by the DSMC.

### Statistical methods

Data are represented as change from baseline, with means and 95% CIs presented for continuous variables, and frequencies and percentages for discrete variables. Baseline data are presented as mean ± SD. The primary analysis of the primary outcome was a covariate-adjusted linear regression estimating the mean group difference (TRE vs IDG) in the change from baseline in HbA_1c_ at 4 months adjusting for baseline HbA_1c_ (continuous), site (Adelaide vs Melbourne) and sex (male vs female; self-reported in the AUSDRISK questionnaire, noting that gender was not asked). Non-inferiority was concluded if the upper 95% two-sided CI of the effect estimate of the difference in HbA_1c_ between groups (TRE – IDG) was less than 1.1 mmol/mol (0.10%). If non-inferiority is concluded, superiority would also be assessed. This primary analysis was for all randomised individuals. Data for those missing their 4 month HbA_1c_ assessment were imputed, with additional complete-case and worst-case sensitivity analyses. Details are provided in the pre-published statistical analysis plan (ESM 3) with one exception. To calculate 24 h glucose iAUC, instead of the proposed baseline CGM algorithm, the method proposed by Chkroun et al [29] was used (ESM 4). In this method, CGM baseline is estimated as the 40th percentile of glucose measures over the period ±24 h, and 24 h glucose iAUC is defined as the area above estimated baseline CGM values.

## Results

### Participants

In total, 247 participants (77% female participants, aged 56±9 years, 32.9 ± 4.3 kg/m^2^) were enrolled in the study; 124 were allocated to TRE, and 123 were allocated to IDG (Fig. 2, Table 1). A similar number of participants were recruited at each site (*n*=128 [52%], Adelaide; *n*=119 [48%], Melbourne).

**Table 1:** Baseline characteristics

Characteristic	TRE (*n*=124)	IDG (*n*=123)
Age, years	57 ± 9	56 ± 9
Female sex, *n* (%)	100 (81)	89 (72)
Place of birth, *n* (%)		
Asia	6 (5)	9 (7)
Australia	92 (74)	80 (65)
Middle East, Northern Africa, Southern Europe	7 (6)	8 (7)
Other	19 (15)	26 (21)
AUSDRISK score^a^	18 ± 3	18 ± 3
Body weight, kg	90.8 ± 14.8	92.5 ± 16.3
BMI, kg/m^2^	33.2 ± 4.3	32.6 ± 4.4
Total fat mass, kg	40.4 ± 8.8	39.9 ± 9.3
Total lean mass, kg	47.3 ± 8.9	49.4 ± 10.0
Waist circumference, cm	108.0 ± 10.7	108.8 ± 11.6
Systolic BP, mmHg	125 ± 13	128 ± 17
Diastolic BP, mmHg	80 ± 6	82 ± 9
HbA_1c_, mmol/mol	40.4 ± 3.3	40.3 ± 3.4
HbA_1c_, %	5.8 ± 0.3	5.8 ± 0.3
Fasting glucose, mmol/l	5.7 ± 0.6	5.6 ± 0.5
Fasting insulin, pmol/l	64.6 ± 40.3	63.9 ± 36.8
HOMA-IR	2.4 ± 1.7	2.4 ± 1.4
CGM metrics^b^		
Time above range, %	3.1 ± 4.5	3.3 ± 4.0
Time in range, %^c^	92.4 ± 9.3	91.2 ± 9.8
Time below range, %	4.5 ± 9.3	5.5 ± 9.9
Nocturnal glucose, mmol/l^d^	5.1 ± 0.6	5.0 ± 0.7
iAUC, mmol/l per 24 h	12.4 ± 3.2	13.2 ± 4.1
SD^e^	0.05 ± 0.02	0.05 ± 0.02
Mean wear time, days^f^	12.1 ± 2.1	11.9 ± 2.4
Total cholesterol, mmol/l	5.5±1.1	5.7 ± 1.3
HDL, mmol/l	1.5 ± 0.4	1.4 ± 0.3
LDL, mmol/l	3.4 ± 0.9	3.6 ± 1.1
Fasting triglycerides, mmol/l	1.5 ± 0.7	1.6 ± 0.9
hs-CRP, mg/l	4.1 ± 6.0	3.4 ± 4.1
Metabolic syndrome, *n* (%)^g^	69 (56)	67 (54)
MEQSA score^h^	59 ± 10	59±8
MEQSA type, %		
Definitely morning type	11	11
Moderately morning type	41	43
Neither type (%)	43	44
Moderately evening type	2	1
Definitely evening type	1	0
Energy intake, kJ/day^i^	9142 ± 2258	9180 ± 2195
Daily eating window, h^j^	11.8 ± 1.22	11.7 ± 1.4
Number of steps per day	8452 ± 2585	8123 ± 2883
METs h/24 h	33.9 ± 1.1	33.7 ± 1.2

Data are shown as mean ± SD or *n* (%)

^a^AUSDRISK assigns a score based on place of birth (by self-report), age, sex, waist circumference, level of physical activity, family history of diabetes, fruit and vegetable intake, BP medication, smoking status, and history of high blood glucose test results (range of possible scores 0–38, where ≥12 or higher as indicative of higher risk of developing type 2 diabetes) [25]

^b^CGM data are from TRE: *n*=117 and IDG: *n*=117

^c^Time in range for this cohort is defined as within ≥3.9 – ≤7.8 mmol/l

^d^Nocturnal mean glucose is derived from CGM glucose between 0000 and 0400 h

^e^Mean across days of the within-day SD

^f^Number of days where CGM data were available

^g^Metabolic syndrome was defined as three of the following: (1) waist circumference of 102 cm for men or 88 cm for women; (2) fasting triglycerides 1.7 mmol/l or drug treatment for elevated triglycerides; (3) fasting HDL-cholesterol of 1.3 mmol/l or drug treatment for reduced HDL-cholesterol; (4) systolic BP 135 mmHg or diastolic BP 85 mmHg or drug treatment for hypertension; and (5) fasting glucose concentrations of ≥5.6 mmol/l [50]

^h^MEQSA is a Morningness–Eveningness questionnaire-self assessment version wherein data are from *n*=122 TRE respondents and *n*=122 IDG respondents scored as follows: definitely morning type, 70–86; moderately morning type, 59–69; neither type, 42–58; moderately evening type, 31–41; and definitely evening type, 16–30

^i^Data are from TRE: *n*=97 and IDG: *n*=106 from Research Food Diary records (5-days)

^j^Data are from TRE: *n*=79 and IDG: *n*=88 from objective food photos

METs, metabolic equivalents from ActivPAL inclinometer per 24 h

### Study conduct

Most of the enrolled participants (>88%) attended all five consultations with no differences between groups (ESM 2/ESM Table 1). At 4 months, 22 (*n*=11 TRE, *n*=11 IDG) participants had discontinued. An additional 34 participants discontinued the trial between 4 and 12 months (*n*=18 TRE, *n*=16 IDG). Between 0–4 months, *n*=4 withdrew due to the TRE intervention and *n*=3 due to the IDG intervention, with the remaining 12 withdrawing due to study burden (*n*=2 from TRE) or unknown reasons (*n*=4 TRE, *n*=6 IDG). Most withdrawals between 4–12 months were due to the intervention (*n*=13 TRE, *n*=7 IDG) or study burden (*n*=4 TRE, *n*=6 IDG). A total of 194 participants completed the 12 month time point (Fig. 2). Protocol violations are detailed in ESM 2/ESM Table 2. Self-reported adherence was higher in the TRE group than in the IDG group at 4 and 12 months (Table 2). In the TRE group, 92% of participants chose a 10:00–19:00 hours eating window, with the remainder choosing 09:00 or 09:30 hours as their starting time. As expected, there was a greater reduction in the reported eating window in the TRE group than in the IDG group at 4 months (estimated change from baseline [95% CI]: −2.6 [−3.0, −2.1] h/day vs −0.6 [−1.0, −0.2] h/day, respectively). There was a greater reduction in self-reported energy intake at 4 and 12 months in the IDG group than in the TRE group (between-group difference [95% CI] 1145 [699, 1591] and 966 [327, 1605] kJ/day, for 4 months and 12 months, respectively) (Table 2). Accordingly, absolute (ESM 2/ESM Table 3) and relative (Table 2) changes in carbohydrate and total and saturated fat were greater in the IDG group than in the TRE group at 4 and 12 months. At 4 months, the relative intake of protein and fibre were increased in the IDG group compared with the TRE group, with relative increased fibre intake persisting at 12 months (Table 2). The reduction in sodium intake was also greater in the IDG group than in the TRE group. There was no difference in alcohol consumption between groups (Table 2).

**Table 2:** Changes in daily eating window, self-reported adherence, dietary intake and ActivPAL-derived metrics after the 4 months intervention and after 12 months

Variable	*n*		Estimated change from baseline (95% CI)		Between-group difference for TRE vs IDG (95% CI)	Between-group *p* value
	TRE	IDG	TRE	IDG		
Adherence and eating window						
Adherence, *n* days/week^a^						
Month 0–4	120	118	5.5 (5.3, 5.8)	4.8 (4.6, 5.0)	0.7 (0.4, 1.1)	<0.0001
Month 4–12	105	104	4.9 (4.6, 5.2)	4.2 (3.9, 4.4)	0.8 (0.4, 1.2)	<0.0001
Daily eating window, h						
Month 4	54	53	−2.6 (−3.0, −2.1)***	−0.6 (−1.0, −0.2)**	−2.0 (−2.5, −1.4)	<0.0001
Month 12	48	44	−2.3 (−2.8, −1.8)***	−0.4 (−0.8, 0.1)	−1.9 (−2.5, −1.3)	<0.0001
Dietary intake						
Energy intake, kJ/day						
Month 4	82	95	−548 (−934, −161)**	−1633 (−1969, −1296)***	1145 (699, 1591)	<0.0001
Month 12	70	77	−811 (−1334, −288)**	−1777 (−2246, −1308)***	966 (327, 1605)	0.003
Carbohydrate, %TEI						
Month 4	82	95	0.4 (−1.0, 1.9)	−1.8 ([−3.1, −0.6)**	2.2 (0.5, 4.0)	0.01
Month 12	70	77	−0.5 (−2.1, 1.1)	−0.8 (−2.2, 0.6)	0.3 (−1.6, 2.3)	0.75
Total fat, %TEI						
Month 4	82	95	0.2 (−1.1, 1.4)	−1.1 (−2.2, −0.1)*	1.3 (−0.2, 2.7)	0.08
Month 12	70	77	0.4 (−1.0, 1.7)	−0.9 (−2.1, 0.3)	1.3 (−0.3, 2.9)	0.11
Saturated fat, %TEI						
Month 4	82	95	−0.02 (−0.7, 0.7)	−1.2 (−1.8, −0.7)***	1.2 (0.4, 2.0)	0.002
Month 12	70	77	0.5 (−0.4, 1.3)	−0.3 (−1.0, 0.4)	0.7 (−0.3, 1.7)	0.15
Protein, %TEI						
Month 4	82	95	−0.1 (−1.0, 0.8)	2.5 (1.7, 3.3)***	−2.6 (−3.7, −1.5)	<0.0001
Month 12	70	77	0.7 (−0.3, 1.6)	1.6 (0.8, 2.4)***	−0.9 (−2.0, 0.2)	0.10
Fibre, %TEI						
Month 4	82	95	−0.04 (−0.2, 0.1)	0.3 (0.1, 0.4)***	−0.3 (−0.5, −0.1)	0.002
Month 12	70	77	−0.02 (−0.2, 0.1)	0.3 (0.1, 0.4)***	−0.3 (−0.5, −0.1)	0.006
Alcohol, %TEI						
Month 4	82	95	−0.4 (−1.2, 0.5)	0.02 (−0.7, 0.8)	−0.4 (−1.4, 0.6)	0.45
Month 12	70	77	−0.4 (−1.3, 0.4)	−0.5 (−1.3, 0.3)	0.06 (−1.0, 1.1)	0.90
Sodium, mg						
Month 4	82	95	−126 (−306, 55)	−375 (−532, −217)***	249 (−36, 463)	0.02
Month 12	70	77	−247 (−432, −62)	−475 (−645, −304)***	228 (−3, 458)	0.04
Eating events, *n*/day						
Month 4	82	95	−2.7 (−3.6, −1.8)***	−2.3 (−3.1, −1.5)***	−0.4 (−1.4, 0.6)	0.45
Month 12	70	77	−2.9 (−4.1, −1.8)***	−2.6 (−3.6, −1.6)***	−0.3 (−1.7, 1.1)	0.72
ActivPAL-derived metrics						
Total number of steps, *n*/day						
Month 4	107	103	−97 (−576, 382)	−160 (−344, 663)	−256 (−826, 313)	0.37
Month 12	81	88	−264 (−878, 349)	−120 (−770, 530)	−144 (−909, 620)	0.71
Total METs h/day						
Month 4	110	104	−0.02 (−0.21, 0.17)	0.05 (−0.14, 0.23)	0.06 (−0.18, 0.30)	0.60
Month 12	86	88	−0.16 (−0.34, 0.03)	0.005 (−0.16, 0.17)	0.16 (−0.07, 0.39)	0.16
Time in bed, h						
Month 4	107	103	0.1 (−0.1, 0.2)	−0.1 (−0.3, 0.04)	0.2 (−0.009, 0.4)	0.06
Month 12	81	88	0.1 (−0.1, 0.3)	−0.2 (−0.3, 0.02)	0.3 (0.04, 0.5)	0.02

Data are presented as change from baseline and mean difference between groups using means and 95% CIs, presented relative to the degree of accuracy it can be measured (i.e. 0.1 h for time, or to the nearest whole number)

Exploratory outcomes were analysed using linear regressions adjusting for the baseline variable and HbA_1c_ (continuous), site (Adelaide or Melbourne) and sex (male or female). The *p* values are two-sided intervention group comparisons of the change in each outcome from these regressions. These analyses were complete-case analyses. Significant within-group effects of time are designated by *p<0.05, **p<0.01, ***p<0.001

^a^Adherence was assessed as described in ESM 3, and reported as average adherence, not change from baseline. Dietary intake and timing outcomes are calculated from objective data collected from Research Food Diary (5 days) and food photos, respectively

%TEI, percentage of total energy intake

### Primary outcome

The results of this study demonstrate that TRE is non-inferior but not superior to IDG for HbA_1c_ at 4 months (mean group difference [95% CI]: −0.22 [−0.72, 0.30] mmol/mol; −0.02 [−0.07, 0.03]%; *p*=0.40) (Table 3). At 12 months, TRE was no longer non-inferior to IDG at the 0.10% margin (mean group difference [95% CI]: 0.47 mmol/mol [−0.19, 1.23]; 0.05 [−0.02, 0.11]%; *p*=0.14). The pre-specified sensitivity analyses resulted in similar conclusions (ESM 2/ESM Table 4). The absolute changes in HbA_1c_ were small in both groups and were not clinically meaningful at any time point (Table 3).

**Table 3:** Primary outcome, and pre-specified secondary outcomes

Outcome	Estimated change from baseline (95% CI)		Mean difference between groups for TRE vs IDG (95% CI)	Between-group *p* value
	TRE	IDG		
Glycaemic markers				
HbA_1c_ at month 4, mmol/mol	−0.40 (−0.83, 0.03)	−0.19 (−0.64, 0.27)	−0.22 (−0.72, 0.30)	0.40
HbA_1c_ at month 4, %	−0.04 (−0.08, 0.003)	−0.02 (−0.06, 0.02)	−0.02 (−0.07, 0.03)	
HbA_1c_ at month 12, mmol/mol	0.12 (−0.47, 0.70)	−0.36 (−0.99, 0.28)	0.47 (−0.19, 1.23)	0.14
HbA_1c_ at month 12, %	0.01 (−0.04, 0.06)	−0.04 (−0.10, 0.02)	0.05 (−0.02, 0.11)	
Secondary efficacy outcomes				
Fasting glucose, mmol/l				
Month 4	−0.09 (−0.18, −0.005)*	−0.12 (−0.22, −0.03)**	0.03 (−0.07, 0.13)	0.56
Month 12	−0.09 (−0.21, 0.02)	−0.10 (−0.21, 0.03)	0.002 (−0.14, 0.14)	0.98
Fasting insulin, pmol/l				
Month 4	−3.33 (−9.24, 2.50)	−9.10 (−15.28, −2.99)**	5.76 (−1.04, 12.57)	0.09
Month 12	−1.81 (−8.68, 5.07)	−7.64 (−14.86, −0.42)*	6.60 (−1.67, 14.79)	0.11
Nocturnal mean glucose, mmol/l^a^				
Month 4	−0.04 (−0.17, 0.10)	−0.05 (−0.19, 0.10)	0.006 (−0.15, 0.16)	0.94
HOMA-IR^b^				
Month 4	−0.03 (−0.12, 0.05)	−0.14 (−0.22, −0.05)**	0.10 (−0.0004, 0.21)	0.06
Month 12	−0.02 (−0.12, 0.08)	−0.10 (−0.19, −0.01)*	0.08 (−0.04, 0.21)	0.18

Data are presented as change from baseline and mean difference between groups using means and 95% CIs

The primary analysis of the primary outcome was a covariate-adjusted linear regression estimating the mean group difference (TRE vs IDG) in the change from baseline in HbA_1c_ at 4 months adjusting for baseline HbA_1c_ (continuous), site (Adelaide or Melbourne) and sex (male or female). This analysis was performed in all randomised individuals using multiple imputations and chained equations within intervention groups and combined using Rubin’s rules. Significant within-group effects of time are designated by *p<0.05, **p<0.01

^a^Nocturnal mean glucose derived from CGM glucose between 00:00 and 04:00 hours

^b^Log transformed data

### Secondary efficacy outcomes

None of the secondary efficacy outcomes (HbA_1c_ at 12 months, fasting insulin, fasting glucose, HOMA-IR and nocturnal mean glucose [by CGM]), differed between groups at 4 and 12 months (Table 3).

### Exploratory outcomes

There was no difference between groups in the reduction in body mass, BMI, waist and hip circumferences, total fat mass, total lean mass and eVAT mass at 4 and 12 months (Table 4). The reductions in total cholesterol and LDL-cholesterol were greater in the IDG group than in the TRE group at 4 months. There were no between-group differences for change in systolic or diastolic BP or in any other cardiovascular markers at 4 and 12 months (Table 4). Time in bed, assessed by ActivPAL, was not different between the two groups at 4 months, although the TRE group had a greater increase in time in bed at 12 months (Table 2). Physical activity (number of steps or total metabolic equivalent h/24 h) was not different between groups at 4 and 12 months (Table 2). Metrics derived from CGM did not differ between groups at 4 months (ESM 2/ESM Table 5). A visual comparison of CGM profiles between baseline and 4 months by group is presented in ESM 2/ESM Fig. 1.

**Table 4:** Changes in cardiovascular markers, body mass and body composition after the 4 month intervention and after 12 months

Exploratory outcome	*n*		Estimated change from baseline (95% CI)		Mean difference between groups (95% CI) for TRE vs IDG	Between-group *p* value
	TRE	IDG	TRE	IDG		
Systolic BP, mmHg						
Month 4	113	111	−2.95 (−5.05, −0.84)**	−2.67 (−4.71, −0.63)**	−0.27 [−2.39, 2.94]	0.84
Month 12	95	95	−2.57 (−4.79, −0.35)*	−1.92 (−4.03, 0.19)	−0.65 [−3.45, 2.15]	0.64
Diastolic BP, mmHg						
Month 4	113	111	−1.81 (−3.02, −0.62)**	−1.40 (−2.57, −0.24)*	−0.41 [−1.92, 1.11]	0.59
Month 12	95	95	−1.15 (−2.38, 0.08)	−0.91 (−2.09, 0.28)	−0.25 [−1.80, 1.31]	0.75
Heart rate, beats/min						
Month 4	108	107	−0.84 (−2.38, 0.71)	−0.90 (−2.39, 0.59)	0.06 [−1.89, 2.02]	0.95
Month 12	92	93	0.55 (−1.01, 2.11)	−1.11 (−2.57, 0.36)	1.66 [−0.29, 3.61]	0.09
Fasting triglycerides, mmol/l^a^						
Month 4	114	111	−0.06 (−0.12, 0.01)	−0.10 (−0.16, −0.04)**	0.04 [−0.04, 0.13]	0.29
Month 12	96	95	−0.04 (−0.11, 0.02)	−0.10 (−0.16, −0.03)**	0.05 [−0.03, 0.14]	0.21
Total cholesterol, mmol/l						
Month 4	114	111	0.07 (−0.10, 0.24)	−0.23 (−0.39, −0.06)**	0.29 [0.08, 0.51]	0.007
Month 12	96	95	0.01 (−0.19, 0.21)	−0.16 (−0.34, 0.03)	0.17 [−0.08, 0.41]	0.17
HDL-cholesterol, mmol/l						
Month 4	114	111	0.02 (−0.02, 0.1)	0.003 (−0.03, 0.04)	0.01 [−0.03, 0.06]	0.59
Month 12	96	95	0.03 (−0.02, 0.08)	0.05 (0.002, 0.09)*	−0.02 [−0.08, 0.04]	0.54
LDL-cholesterol, mmol/l						
Month 4	114	111	0.13 (−0.01, 0.27)	−0.13 (−0.27, 0.002)*	0.26 [0.08, 0.44]	0.004
Month 12	96	95	0.07 (−0.09, 0.22)	−0.10 (−0.25, 0.04)	0.17 [−0.02, 0.36]	0.08
hs-CRP, mg/l^a^						
Month 4	114	111	0.06 (−0.11, 0.23)	−0.11 (−0.27, 0.05)	0.17 [−0.04, 0.38]	0.10
Month 12	96	95	0.007 (−0.18, 0.19)	−0.16 (−0.33, 0.02)	0.16 [−0.07, 0.40]	0.16
Body mass, kg						
Month 4	113	111	−2.05 (−2.69, −1.41]***	−2.36 (−2.98, −1.74]***	0.31 [−0.49, 1.10]	0.44
Month 12	95	95	−2.02 (−3.08, −0.95)***	−2.91 (−3.92, −1.90)***	0.89 [−0.43, 2.22]	0.18
BMI, kg/m^2^						
Month 4	113	111	−0.73 (−0.95, −0.51)***	−0.81 (−1.02, −0.60)***	0.08 [−0.19, 0.36]	0.54
Month 12	95	95	−0.71 (−1.08, −0.33)***	−1.03 (−1.38, −0.67)***	0.32 [−0.15, 0.79]	0.18
Waist circumference, cm						
Month 4	112	111	−2.88 (−4.03, −1.73)***	−2.84 (−3.95, −1.73)***	−0.04 [−1.48, 1.40]	0.95
Month 12	95	95	−2.24 (−3.68, −0.80)**	−2.99 (−4.36, −1.62)***	0.75 [−1.06, 2.55]	0.41
Hip circumference, cm						
Month 4	112	111	−2.01 (−2.88, −1.13)***	−2.55 (−3.40, −1.71)***	0.55 [−0.56, 1.65]	0.32
Month 12	95	95	−2.54 (−3.64, −1.44)***	−3.22 (−4.25, −2.18)***	0.68 [−0.70, 2.05]	0.33
Fat mass, kg						
Month 4	112	108	−1.43 (−1.96, −0.91)***	−1.88 (−2.39, −1.37)***	0.45 [−0.22, 1.11]	0.18
Month 12	86	88	−1.36 (−2.28, −0.45)**	−2.30 (−3.16, −1.44)***	0.94 [−0.22, 2.10]	0.11
Lean mass, kg						
Month 4	112	108	−0.54 (−0.80, −0.28)***	−0.28 (−0.54, −0.03)*	−0.25 [−0.55, 0.04]	0.09
Month 12	86	88	−0.61 (−0.96, −0.26)***	−0.65 (−0.98, −0.32)***	0.04 [−0.37, 0.44]	0.86
eVAT mass, kg						
Month 4	112	107	−0.09 (−0.15, −0.03)**	−0.12 (−0.17, −0.06)***	0.02 [−0.05, 0.09]	0.51
Month 12	86	88	−0.07 (−0.15, 0.02)	−0.13 (−0.20, −0.05)**	0.06 [−0.04, 0.16]	0.23

Data are represented as change from baseline and mean difference between groups using means and 95% CIs

Exploratory outcomes were analysed using linear regressions adjusting for baseline HbA_1c_ (continuous), site (Adelaide or Melbourne) and sex (male or female). These analyses were complete-case analyses. Significant within-group effects of time are designated by *p<0.05, **p<0.01, ***p<0.001

^a^Log transformed data

### Adverse events

The most frequently reported adverse events were flu-like symptoms, fatigue, infection, pain, diarrhoea, falls, headache, nausea/vomiting and presyncope. Frequency and type of adverse events were not different between interventions (ESM 2/ESM Table 6).

## Discussion

This low-oversight clinical trial demonstrated that TRE was non-inferior but not superior to IDG delivered via telehealth on HbA_1c_ at 4 months in individuals at risk of developing type 2 diabetes. However, at 12 months, the treatment no longer satisfied the non-inferiority criterion set at 1.1 mmol/mol (0.10%), meaning the difference between groups was too large to conclude that TRE was non-inferior to IDG. Both interventions did modestly reduce body weight and improve body composition and BP, with the effects being sustained over 12 months. Self-reported adherence to the intervention was higher with TRE, suggesting greater acceptability over 12 months. The study highlights TRE as a pragmatic short-term alternative to individualised dietary guidance, which could be implemented where access to a dietetics service is limited, or if the individual preferred such an approach.

To test how TRE might be utilised in a primary care setting, we chose to mimic the existing chronic disease management plan in Australia, whereby individuals can access up to five subsidised dietetic consultations in a calendar year [32]. Within this five-visit telehealth approach, TRE was not inferior to IDG on HbA_1c_ at 4 months. However, the absolute changes in HbA_1c_ were small and not clinically meaningful in either group at either time point. A meta-analysis showed that lifestyle interventions with 12–26 sessions in a calendar year result in body mass losses of ~6% over 12 months, whereas those with less than 12 sessions result in ~2.8% loss [33]. A systematic review and meta-analysis of lifestyle interventions between 3 and 12 months in duration showed reductions in HbA_1c_ of ~1.6 mmol/mol (0.15%) in people at risk of type 2 diabetes [34]. The effectiveness of lifestyle interventions are more modest when studied in primary care or telehealth settings [35, 36], which tend to be limited by the number of support sessions available.

In a population at elevated risk of type 2 diabetes, delaying or preventing a rise in HbA_1c_ is integral for prevention and progression to disease. While we enriched the population by enrolling individuals with an elevated AUSDRISK score, which correlates with increased type 2 diabetes risk [25], less than one-third of our cohort would be classified as living with pre-diabetes (i.e. HbA_1c_ >39 mmol/mol [5.7%] [37]). The inclusion of a more clinically ‘healthy’ population at baseline could explain the lack of efficacy of both interventions for improving HbA_1c_. A pragmatic trial in 54 adults with at least one component of the metabolic syndrome compared TRE against standard dietary advice and found no differences in fasting glucose concentration and HbA_1c_ after 6 months [38]. Combining 8 h early (10:00–18:00 hours), late (13:00–21:00 hours) or self-selected TRE with a Mediterranean diet resulted in greater weight loss as compared with the Mediterranean diet alone in 197 individuals with overweight and at least one marker of the metabolic syndrome. However, there were no differences in glycaemic outcomes, except for lower fasting glucose concentrations in the early TRE group, likely due to a longer fasting period [39]. In contrast, a 0.1% greater reduction in HbA_1c_ was observed in a combined TRE plus Mediterranean diet intervention over Mediterranean diet alone in 108 adults with metabolic syndrome [23]. In populations with insulin resistance or pre-diabetes, 6 h TRE improved fasting insulin and HOMA-IR, but did not alter HbA_1c_, over 4–8 weeks [6, 9, 12]. A recent systematic review and meta-analysis found that TRE improved HbA_1c_ and fasting insulin concentrations, albeit with high heterogeneity among studies [19]. Collectively, it is inconclusive whether a TRE intervention delays or prevents a rise in HbA_1c_.

Most previous TRE interventions have allocated change in body mass as the primary outcome [15, 17, 40–42]. In our study, both interventions reduced body mass (~–2.5 kg) and improved body composition after 4 months. The loss in body mass is comparable with that reported in other studies of TRE with similar eating windows and intervention duration [13, 23] and likely related to a small reduction in energy intake (~400 kJ/day [95 kcal/day]) as a result of a 2.5 h reduction in eating window. Unlike the IDG group, the TRE group received no advice to reduce portion sizes or improve diet quality and only received advice around meal timing. The IDG group were counselled on dietary quality and were asked to record their dietary intake for 3 days prior to each consultation, likely improving their awareness of food choices. There was a larger reduction in self-reported energy intake in the IDG group but this was not mirrored by greater body mass loss, and thus we suspect a greater degree of under-reporting occurred as a result of the knowledge gain.

Importantly, the modest reductions in body mass were retained after 12 months in both groups. A pooled data analysis by Yaskolka Meir and colleagues reported that every 1 kg of sustained weight loss from lifestyle interventions was associated with improved lipid markers (+1.4% HDL-cholesterol, −1.4% triglycerides) and insulin resistance (insulin −2.5%, HOMA-IR −2.7%) [43] demonstrating the importance of maintaining body mass loss. The longevity of body mass loss may be related to the intervention delivery, where both groups received behavioural tools designed to educate and incorporate into daily routines. The finding that TRE resulted in a similar body mass loss to IDG without dietary counselling or prescribed energy restriction highlights the potential for TRE as a practical weight management tool that could be implemented with minimal support from healthcare providers to initiate behaviour change.

Dietetic guidance is a well-established intervention to improve dietary quality, which impacts markers of cardiometabolic health [44, 45]. In the current study, IDG induced greater improvements in diet quality and produced greater improvements in total and LDL-cholesterol as compared with TRE, with the TRE group being instructed solely to modify the timing of their energy intake. Taken collectively, our findings suggest that TRE could serve as a practical interim strategy to induce modest body mass loss and reductions in BP, although gradual integration of dietary advice appears necessary to elicit meaningful improvements in blood lipids.

The TRE group had higher self-reported adherence rates at 4 and 12 months compared with the IDG group. Higher rates of adherence have previously been reported in TRE over control [10] or energy restriction [21]. The greater adherence may be reflective of the ease of implementation, since participants are not required to acquire additional nutrition knowledge or make major changes to food choices or purchasing habits, reducing cognitive load and decision-making demands. The TRE timing of ~10:00 to 19:00 hours (which ~92% of participants chose) results in a delayed breakfast and earlier dinner than typical Australian meal patterns [46]. In contrast to other interventions [15, 23, 39], participants were not required to provide a daily log of dietary intake or eating windows to simulate real-world conditions with minimal participant accountability and enhanced the ecological validity of the intervention. Despite there being greater self-reported adherence to the TRE intervention, this did not translate to greater improvements in health markers, suggesting that the nuances around timing are not as holistically effective as traditional dietary guidance and that self-reported adherence may not indicate true adherence.

The strengths of this study are the cohort size, the largest to date, providing sufficient power to test non-inferiority of changes in our primary outcome of clinical glycaemic status via HbA_1c_. Further, our utilisation of telehealth demonstrates the potential for implementation of the intervention for individuals living in rural or remote settings, who typically have limited access to dietetics and healthcare services and poorer social determinants of health [4, 47, 48]. Except for dietetic consultations over the first 3 months, the study was delivered with minimal participant contact, enabling TRE to be tested using a real-world approach, that could serve as evidence for implementation in primary healthcare settings as an initial dietary strategy. To facilitate greater acceptability, a conservative time restriction of 9 h was selected. While it is plausible that a shorter eating window may have induced greater improvements in several outcomes, such approaches have not been tested beyond 3–4 months and the long-term sustainability of a more stringent intervention remain unclear. As with any dietary study, the accuracy of the data reflects the accuracy of the participants’ reporting, which is likely one of the reasons why many TRE studies do not report dietary intake [49]. Additionally, like most dietary intervention studies, the majority (~75%) of our cohort were female, predominantly white, and we did not capture socioeconomic status, limiting the full extrapolation of our findings. Our rigorous assessment of the dietary data [20] ensured the analysis of participant-reported intake was as accurate as possible, but this does not fully account for mis- or under-reporting by participants.

### Conclusion

When delivered remotely and with consultations aligned with a Medicare Chronic Disease Management Plan, TRE was not inferior to IDG for HbA_1c_ over 4 months, although neither intervention improved HbA_1c_. Both strategies induced modest weight loss and BP improvements that were maintained for 12 months. TRE showed greater acceptability than IDG and thus may be an alternative strategy, particularly when access to dietetic services is limited.

## Supplementary Information

Below is the link to the electronic supplementary material.

ESM (PDF 3120 KB)

## Data Availability

Anonymised data from this study are available on request from the corresponding author for 36 months from date of publication with a full research plan for academic use only. The data are not publicly available as they contain information that could compromise research participant consent.

## Abbreviations

APD: Approved Practising Dietitian
AUSDRISK: Australian type 2 diabetes risk assessment tool
CGM: Continuous glucose monitoring
DSMC: Data and Safety Monitoring Committee
eVAT: Estimated visceral adipose tissue
hs-CRP: High-sensitivity C-reactive protein
iAUC: Incremental AUC
IDG: Individualised dietetic guidance
MET: Metabolic equivalent
TRE: Time-restricted eating
