# Supplementary material for: Time-restricted eating versus dietetic guidance on glycaemic outcomes in adults at risk of type 2 diabetes: a non-inferiority randomised clinical trial
Source: Diabetologia. 2026 Jun 6;69(9):2458–71. doi: 10.1007/s00125-026-06762-x (PMC13424568; doi:10.1007/s00125-026-06762-x)
Supplement: Supplementary file 1 — ESM (PDF 3120 KB) [file 125_2026_6762_MOESM1_ESM.pdf]

Electronic Supplementary Material: ESM 1

HUMAN ETHICS COMMITTEE PROTOCOL APPLICATION

CENTRAL ADELAIDE LOCAL HEALTH NETWORK

1. Title: Time **R**estricted **EAT**ing to reduce the risk of type 2 diabetes (TREAT)

2. Investigator details

a) Associate Professor Leonie Heilbronn, BSc (Hons), PhD. Adelaide Medical School, University of Adelaide. *Principal Investigator.* leonie.heilbronn@adelaide.edu.au
b) Dr Amy Hutchison, BSc (Hons), PhD. Adelaide Medical School, University of Adelaide. *Co-investigator, Postdoctoral Researcher, Clinical trial co-ordinator* (University of Adelaide, South Australian Health and Medical Research Institute site). amy.hutchison@adelaide.edu.au
c) Professor John Hawley, BSc (Hons), Cert. Ed., M.A., PhD. Exercise and Nutrition Research Program, Mary Mackillop Institute for Health Research, Australian Catholic University. *Co-investigator; site lead, ACU.* john.hawley@acu.edu.au
d) Dr Evelyn Parr, BPhEd, BSc, MPhEd, PhD. Exercise and Nutrition Research Program, Mary Mackillop Institute for Health Research, Australian Catholic University. *Co-investigator, Postdoctoral Researcher, DXA technician.* evelyn.parr@acu.edu.au
e) Dr Brooke Devlin, BExSci, MNutrDiet, PhD. Department of Dietetics, Nutrition and Sport, La Trobe University. *Co-investigator, Dietetic oversight/lead dietitian.* b.devlin@latrobe.edu.au
f) Associate Professor Leah Brennan, BAppSc (Hons), MSc, PhD. School of Psychology and Public Health, La Trobe University. *Co-investigator, Psychological oversight/study psychologist.* l.brennan@latrobe.edu.au
g) Professor Gary Wittert MBBS, PhD, FRACP. Adelaide Medical School, University of Adelaide. Senior Consultant Endocrinologist, Royal Adelaide Hospital. *Study Physician / medical oversight.* gary.wittert@adelaide.edu.au
h) Dr Andrew Vincent, PhD. University of Adelaide. *Statistician.* andrew.vincent@adelaide.edu.au
i) Ms Rasha Charrouf, MSc. Adelaide Medical School, University of Adelaide. *PhD Student.* rasha.charrouf@adelaide.edu.au
j) Dr Bo Liu, MMedSci, PhD. Adelaide Medical School, University of Adelaide. *Postdoctoral Researcher.* b.liu@adelaide.edu.au
k) Mr Bradley Klingner, BSc. CSIRO. *DXA technician, Adelaide site* bradley.klingner@csiro.au.
l) Mr Steve Flint, B.Ex. Sc. (Hons), M. Diet, APD. Exercise and Nutrition Research Program, Mary Mackillop Institute for Health Research, Australian Catholic University. *Study dietitian.* Steve.flint@acu.edu.au

m) Ms Bridget Radford, B. Hum. Nut. (Hons). Exercise and Nutrition Research Program, Mary Mackillop Institute for Health Research, Australian Catholic University. *Research Assistant, DXA technician.* bridget.radford@acu.edu.au
n) Ms Rebecca Hall, B. Nut. Diet. (Hons), APD. Exercise and Nutrition Research Program, Mary Mackillop Institute for Health Research, Australian Catholic University. *Research Assistant, Dietitian, DXA technician.* rebecca.hall@acu.edu.au

3. Introduction

Intensive nutrition and physical activity interventions can delay or prevent the development of type 2 diabetes (T2DM) in at-risk individuals by up to 58% over 10 years [1]. However, long-term adherence to lifestyle modifications that restrict energy intake continues to be poor [2]. Recent research shows that erratic and poor timing of meals is a contributing factor to metabolic disease risk, including T2DM [3, 4]. This means that **when** we eat could be as, or more, important as *what* we eat for metabolic health. Work conducted by this team suggests that time restricted eating (TRE), whereby individuals are instructed to confine all energy intake to 8-10 h per day, is a practical intervention that reduces glycated haemoglobin (HbA1c) and the glucose and insulin responses to meals in people with obesity and/or T2DM, at least in the short-term (1-12 weeks). Importantly, TRE improved insulin sensitivity, blood pressure and oxidative stress, independent of any weight loss [5]. The proposed multi-centre randomised clinical trial will be a world-first and will determine the long-term effects of TRE on glucose control and cardio-metabolic health at 4 and 12 months follow up. Based on our extensive preliminary data, *we hypothesise that TRE will be at least as effective as current dietetics practice guidelines (CP) to improve glycaemic control in individuals at high risk of developing T2DM.* The impacts of TRE on body mass and body composition, markers of cardiovascular disease (CVD), wellbeing, quality of life and enablers and barriers to adherence will also be examined. Given the simplicity and practicality of TRE as a dietary intervention, we anticipate TRE will be associated with greater adherence and satisfaction at 12 months.

4. Background

There has been a sharp decline in the number of individuals who report eating three meals a day, with an increase in prolonged eating patterns [6, 7]. Australian data from 24-h dietary recalls indicates that a 'grazing' eating pattern reduces dietary quality and increases intake of discretionary foods [8]. Eating patterns were also tracked using a smartphone application in the USA in 156 non-shift workers for 3 weeks [9]. That study revealed that more than 50% of their cohort ate erratically and had shortened overnight fasting (9-10 hours) periods [9]. The duration over which food is consumed during a day (i.e., the "feeding-fasting cycle") has marked effects on a number of physiological and metabolic processes, independent of total energy intake and/or food content [5, 10]. Evidence to support this premise came initially from studies undertaken in mice and fruit flies, in which access to food was "restricted" by increasing the time spent fasting: this was compared to an unrestricted or free-living eating schedule (12-14). In such studies TRE restored circadian amplitude, protected mice against high-fat diet-induced

obesity, hyperinsulinemia, hepatic steatosis, and inflammation - despite no reductions in energy intake [10]. These results are striking, but there are many physiological and metabolic differences between small model organisms and humans [11]. Overall, the current evidence shows that TRE improves glycaemic control and/or reduces CVD risk factors, and induces modest reductions in body weight (2-3 kg). To date, all human intervention studies have been short-term (4 days to 12 weeks), with small sample sizes (8 - 40 individuals), and for the most part limited to men. Only five studies have compared TRE to a control group and none have compared to a group that is receiving best dietary practice guidelines.

Preliminary data: The CIs have performed four pilot studies showing the acute glycaemic benefits of TRE in individuals at risk of, or with overt T2DM, which have informed the proposed study design.

Utilising a randomised, cross-over design the CIs at ACU investigated the effects of 5-d of TRF (8-h/day) or extended feeding (EXF) in 11 individuals with overweight/obesity (BMI 32 ± 2 kg/m^2). Participants completed two isoenergetic diets for 5 days each as three identical meals at either 10 am, 1 pm and 5 pm (TRF; 8 h) or at 7 am, 2 pm and 9 pm (EXF; 15 h). On the 5th day, participants spent 24 h in a clinical ward. Blood was sampled at regular intervals for analysis of glucose and insulin concentrations. TRE reduced nocturnal glucose levels and tended to improve glucose and insulin profiles over 24-h as compared to unrestricted (15 h) feeding [12]. They recently conducted a 4-week feasibility study of TRE (9-h/day, 1000-1900 h) in individuals with T2DM (HbA1c < 9%). Prior to a 2-week habitual period and following 4-weeks of TRE (self-reported adherence 5 d/wk), 19 participants completed a mixed-meal tolerance test (MMTT; 50% CHO, 30% fat, 20% protein) and HbA1c testing. Glucose AUC showed a trend to be lower after TRE (P=0.09, Fig. 3) while HbA1c was significantly reduced (-0.24%; P=0.05).

The CIs at SAHMRI conducted a free-living randomised cross-over trial in 15 men with obesity [13]. One week of TRE (9-h/day) initiated early (eTRE 0800-1700 h) or TRE initiated with a phase delay (dTRE 12 *pm* – 9 *pm*) significantly improved glucose tolerance by 38% (eTRE) and 40% (dTRE), respectively. This study showed there is some flexibility in the clock time that TRE can be initiated. They recently completed a study examining the effects of 8 weeks of TRE (10-h/day, self-selected window - except the last intake must have occurred prior to 1930 h) on glycaemic control in 15 men with obesity (BMI 31.0 ± 2.5 kg/m^2, Waist 113 ± 9 cm, Age 63 ± 5 y). This study was free-living except for a 3 day lead in period prior to and during a 35 hour stay on a metabolic ward when foods were provided at calculated energy requirements and strictly timed (3 identical meals and 2 snacks provided over 14 h at W0 and 10 h at W8). TRE significantly reduced body weight (-2.3 ± 0.6 kg, P=0.03), HbA1c (Figure 2A) and glucose AUC to breakfast (Fig 2B). Adherence to TRE was 5.9d per week (measured by smartphone app) and participants displayed reduced eating occasions and reduced variability in eating behaviour, which is linked with reduced disease risk.

5. Purpose

The proposed study will be the first randomised, controlled trial to determine the long term efficacy, feasability and acceptability of TRE in a large cohort of men and women at high risk of developing T2DM.

Aim: To conduct a multi-centre randomised controlled trial to compare time-restricted eating (TRE) with current best practice (CP) guidelines in dietetics on glycaemic control and metabolic parameters over 12 months, in individuals at high risk of T2DM.

Primary Outcome: Change in glycated haemoglobin (HbA1c) at 4 months.

Secondary Outcomes: Change in HbA1c at 12 months; Change in fasting glucose, insulin, C-reactive protein, blood lipids and blood pressure, changes in novel cardiovascular risk markers, changes in advanced glycation endproducts, changes in inflammatory markers, changes in plasma/serum proteome, changes in plasma/serum metabolome, changes in telomere length, changes in urinary oxidative stress markers, changes in urinary markers of diet quality, 24-h profiles of glycaemia by CGM (FreeStyle Libre Pro); body mass and body composition by DXA; physical activity by inclinometer (ActivPAL), adherence by EasyDietDiary (EDD) smartphone application (app); mood, diet quality, food preferences, quality of life, sleep assessed by questionnaire and barriers and enablers to adherence by semi-structured interview; at 4 and 12 months.

Hypothesis: We hypothesise that TRE will not be inferior to current practice guidelines (CP) in dietetics to reduce HbA1c in individuals at high risk of developing T2DM.

6. Study design

Setting: A parallel, single-blinded, multi-centre randomised controlled trial conducted at the South Australian Health and Medical Research Institute (SAHMRI) and the Mary Mackillop Institute for Health Research (MMIHR), Australian Catholic University, by researchers from the University of Adelaide, Australian Catholic University and La Trobe University.

Summary: In a parallel groups design, a total of 268 individuals will be recruited across both sites. After collection of baseline characteristics, participants will be randomised into one of two groups (TRE, time-restricted eating; CP, current practice guidelines). All participants will receive 5 telehealth consultations with an Accredited Practising Dietitian (APD) at the specified time points below, the content of which will be based around their randomised condition. They will undergo metabolic testing on 2 further occasions (4 months, 12 months) over a 12-month period (Figure 1) to assess the changes in primary and secondary outcomes (detailed above). Due to the nature of this intervention, neither participants nor clinical research staff or dietitians can be blinded to group assignment, but all laboratory work and data analysis will be performed blinded.

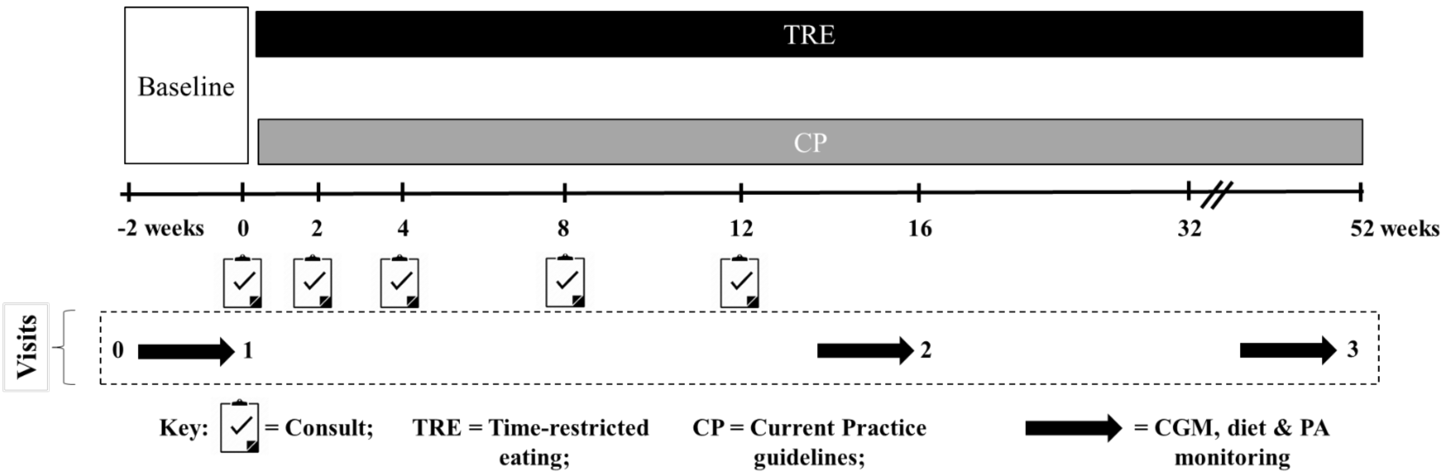

Participants:

Study population: 268 men and women will be recruited from the greater metropolitan areas of Adelaide and Melbourne.

Inclusion criteria: Study participants will be aged 35 to 70 years, overweight or obese (BMI: >25 but <45 kg/m^2), and will score ≥15 on the AUSDRISK assessment tool (attachment 4: questionnaires).

Exclusion criteria include:

A personal history/diagnosis (self-reported) of:

- diabetes (type 1 or 2)
- major psychiatric disorders (schizophrenia, major depressive disorder, bipolar disorder, eating disorders)
- gastrointestinal disorders/disease (including malabsorption)
- haematological disorders (i.e. thalassemia, iron-deficiency anaemia)
- insomnia
- currently receiving, or have received treatment/diagnosis of cancer in the past 3 years (excluding non-melanoma skin cancer)
- significant liver or kidney disease requiring ongoing medical care
- previous or planned gastro-intestinal surgery (including bariatric surgery)
- Congestive heart failure (NYHA stage 2 or above)
- Previous myocardial infarction or significant cardiac event ≤ 6 months prior to screening
- Previous cerebrovascular event ≤ 12 months prior to screening

and/or any other condition deemed unstable by the study physician.

Currently taking the following medications:

- any medication used, or known to lower blood glucose, or antidiabetic medications, including, but not limited to: SGLT2 inhibitors, metformin, sulfonylureas, glucagon-like peptide-1 (GLP-1) analogues [i.e. exenatide], thiazolidinediones or DPP-IV inhibitors [i.e. 'gliptins'])
- Medications affecting weight, appetite or gut motility, including, but not limited to: (domperidone, cisapride, orlistat, phentermine, topiramate).
- Diuretics (i.e. frusemide, thiazides) or combination blood pressure medications containing a diuretic
- Beta-blockers
- Glucocorticoids
- Anti-epileptic medications
- Antipsychotic medications
- Opioid medications unless combined with paracetamol in a single formulation and used occasionally on a PRN basis

Participants who are taking stable doses (i.e. > 3 months) of androgenic medications (i.e. testosterone), blood pressure medications (not listed above) or lipid medications, thyroid medication, or SSRI's will ***not*** *be excluded.*

Additional exclusion criteria include:

- do not consume a regular breakfast (i.e. eat breakfast on an average of 5 or more days per week), OR do not eat for more than 12 hours per day on an average of 5 or more days per week
- have an extreme or restricted pattern of eating (i.e. following an intermittent fasting diet) or already engaged in a TRE protocol
- shift-workers
- pregnant, planning a pregnancy or currently breastfeeding
- those who have lost or gained >5% of body weight in the last 6 months
- alcohol intake of greater than 10 standard drinks per week, or more than 4 standard drinks on any one day (as per NHMRC draft guidelines)
- current smokers of cigarettes/marijuana/e-cigarettes/vaporisers
- anyone unable to comprehend the study protocol or provide informed consent (i.e. due to English language or cognitive difficulties)
- Participants will not have seen a dietitian in the preceding 3 months.

Recruitment: The study will be advertised through The University of Adelaide, La Trobe University and Australian Catholic University networks, local newspapers and social media, and using a third-party trial recruitment company. The inclusion criteria will be included in the advertising material, and participants will self-identify as potentially eligible. On occasion, researchers in our groups have been approached by local media (radio, newspaper, television)

to comment on our current research. These articles are sometimes used to recruit participants, by providing a phone number or email address whereby participants can contact the researchers to express an interest in taking part.

Upon registering their interest, participants will be provided with a copy of the participant information sheet (PIS; attachment 1: Participant information sheet), and allowed as much time as they require to decide whether they would like to participate. They will also be asked to complete an online screening questionnaire via RedCAP which gathers demographic and work-related information, assesses the 11 AUSDRISK criteria, and collects diet, medical and exercise history.

Existing databases of participants who have previously registered their interest to take part in research at each site will also be utilised. Potentially eligible participants will be identified and sent a generic email by the Trial Coordinator at each site (attachment 3: generic email) notifying them of the study, and asking for expressions of interest. A copy of the PIS, and a secure link to the screening questionnaire will also be included.

Informed consent: After completion of the screening questionnaire, potentially eligible participants will be invited to attend either South Australia Health & Medicine Research Institute (Adelaide) or the Mary MacKillop Institute for Health Research at Australian Catholic University (Melbourne), and will have the research protocol explained to them in detail by a senior member of the research team. They will be provided with an opportunity to ask questions. Informed consent, including a verbal indication that they understand the general study protocol and requirements will then be obtained, prior to any further testing (attachment 2: consent form). After consent is obtained, weight, height and waist circumference, using standardised procedures, will be assessed to ensure suitability for inclusion into the trial. A finger prick blood sample will be collected to assess baseline HbA1c, and to screen for undiagnosed diabetes. An HbA1c ≥6.5% will be deemed ineligible, and participants will be referred to their GP for follow-up. Participants will also be asked to complete a Kessler Psychological Distress scale (K10) and eating disorders examination questionnaire (EDEQ), to exclude participants with an eating disorder or major depression. A score ≥30 on the K10 and/or ≥2.8 on the EDEQ (1 SD above community norms in Australia for young women and used for ‘remission/recovery’ in clinical trials) will be utilised. Any participants who are deemed ineligible on this basis will be referred to their GP for follow-up. If participants meet the initial eligibility criteria, they will be invited to take part in the remainder of the study.

Methodology:

Enrolment: Eligible participants who provide informed consent and enrol into the study will be invited to return for a baseline visit (V1), where they will be fitted with a continuous glucose monitor (CGM; Freestyle Libre Pro), an inclinometer (Actipal) and instructed how to use a validated “Easy Diet Diary” (EDD) smartphone app to simultaneously record habitual levels of glycaemia, activity and dietary intake/eating patterns, respectively, for 2-weeks. Handwritten recordings that include times that food was eaten will be used if participants are not comfortable using the app. Participants will complete a series of questionnaires (detailed

below in *data collection*), anthropometric measurements will be taken, and a DXA scan completed to assess body composition. A 40 mL blood sample and spot urine sample will be collected from participants.

After completing V1, participants will be randomised to one of two groups for 12 months. All participants will undergo identical measurements including dietary recordings and both groups will receive five individual dietary consultations via telehealth (i.e. same amount of contact time) with an Accredited Practising Dietitian (APD at 0 [V1], 2, 4, 8 and 12 weeks). At V1, both groups will also be encouraged to increase physical activity and will receive information and guidance in booklet format "Australia's Physical Activity & Sedentary Behaviour Guidelines for Adults (18-64 years)".

Randomisation: Stratified random length blocked randomisation by site and baseline HbA1c will be under direction of Dr Vincent who will have no contact with participants.

Sample size calculation: In a population of individuals with pre-diabetes, the clinical difference in HbA1c that is considered not to be relevant is 0.2% [14]. Literature suggests that the within group standard deviation to be 0.6% in this population of pre-diabetics [14, 15]. Assuming that attrition at 4 months is less than 20% and the pre vs post HbA1c correlation is 0.5, then with n=268 randomized equally to each intervention (134 each arm), there is 80% power to conclude that TRE is non-inferior to CP with a margin of 0.2% in a baseline adjusted ANCOVA when the true difference is zero (2-sided alpha=0.05).

Intervention diets

Time-Restricted Eating (TRE): The TRE group will be instructed to follow TRE (9 h/day) every day for 12 months with no other dietary instructions or advice provided. The TRE group will attend the same consult schedule with an APD as the CP group, but consultations will focus on **timing** of dietary intake and strategies to promote adherence. No dietary guidance regarding quantity or quality will be provided. Participants will be able to self-select the precise 9-h schedule that will best suit their lifestyles, with the caveat that the latest time of eating will be set at 7:30 pm. Outside of the elected eating window, participants will be allowed to consume water and black coffee and/or tea.

Current Best-Practice guidelines (CP): This group is designed to act as a comparator using 'standard care' in dietetics practice. Dietary advice provided to this participant group will be performed by APDs in line with evidence-based guidelines; T2DM best-practice guidelines plus Australian Dietary Guidelines (i.e. Australian Guide to Healthy Eating) [16] to improve **diet quality,** and strategies to promote adherence. No specific advice will be provided regarding time of day to start and finish eating and/or drinking (since this information is not outlined in current practice guidelines).

Dietary consultations

The consults are based around the five dietitian visits that individuals who meet the criteria for a Chronic Disease Management Plan are eligible for, and will occur at the end of the habitual

period (week 0), 2, 4, 8 and 12 weeks into the intervention. The consultations will be conducted via telehealth by the study dietitians. At each telehealth appointment, a dietitian will consult with each participant individually to provide guidance on timing of eating (TRE) or dietary changes to improve quality of intake (CP). Participants will be asked to set individual goals at each session which will be used as focal points for subsequent visits, to assist with having directed changes and maintaining motivation (as per usual dietetic practice). Advice is tailored to cultural and personal preferences. Importantly, both groups will have identical visit schedules and measures of adherence. Prior to each diet consult, participants will also be required to use the EDD app to log all energy intakes, with photos, for 5 days. This will be used as a tool by the dietitian to provide individualised advice and assess compliance. After the 16 week metabolic visit, dietetic support will be withdrawn, and participants will be encouraged to continue their respective interventions.

Data collection: The following sections outline the methods of data collection. These methods are established at both sites, and standardised to a single protocol.

Metabolic assessments

Each of the three metabolic data collection visits are standardised and routinely performed in the clinic research facilities. Participants will arrive at the clinic at ~0730 h after an overnight fast (from 1930 h for all groups). Body composition by GE Lunar iDXA is assessed, including body mass (gowned), waist and hip circumference, and blood pressure (seated 10 min rest). Fasting blood samples (40 mL) will be taken. Blood samples are centrifuged immediately, and snap frozen at -80 ºC for later assessment of hormones, peptides and metabolites. Visual analogue scales are administered to assess perceived satisfaction with the dietary program. At each data collection visit (V1-3), lifestyle, health and well-being, sleep timing and quality and food intake behaviours will be assessed using a series of validated questionnaires (Assessment of Quality of Life [17], Diet Related Quality of Life Scale (currently being developed by Dr Devlin and A/Prof Brennan; the questionnaire is comprised of 207 items in a Likert scale format (1 = *Always true* to 5 = *Never true*)), Depression, Anxiety and Stress Scale [DASS] [18], Eating Disorders Examination Questionnaire [EDEQ] [19], Clinical Impairment Scale [20], Pittsburgh Sleep Quality Index [PSQI] [21], Horne and Östberg's Morningness-Eveningness questionnaire [22]; (attachment 4: questionnaires). Qualitative semi-structured interviews about the participant’s experience and satisfaction will be conducted and audio-recorded at 4 and 12 months to ascertain the enablers and barriers to adherence. An adherence questionnaire will also be completed.

Laboratory testing: All analytes will be assessed at SAHMRI using standard kits/methods that are all established in CIAs laboratory. Briefly, blood samples are collected, processed immediately, and snap frozen at -80 °C. Samples are analysed for HbA1c, glucose, triglyceride, total cholesterol, HDL-cholesterol, non-esterified free fatty acids, alanine aminotransferase, C-reactive protein on auto-analysers using standard enzymatic kits (Roche Diagnostics, WAKO, Sigma-Aldrich). Insulin concentration is measured by ELISA. Urinary isoprostanes will be assessed by ELISA to quantify oxidative stress. Buffy coat will be used to assess changes in telomere length.

Adherence to allocated dietary intervention: Both groups will upload photographs of, and record all daily energy intake using the EDD app (in which participants will be trained) through the Habitual baseline period and for two weeks prior to each of the data collection visit (i.e. V1-3). Files are automatically imported to Foodworks 9.0 (Xyris, Australia) to calculate energy and macronutrient intake. Adherence to the assigned group will be assessed from diet/photo records. They will also be asked to log all activity during each diet recording period, using the EDD app (in addition to data from the Activpal monitors). At each dietitian consult, and once every 4 weeks between week 16 and 52, participants will be asked to complete a Likert scale assessing their self-reported adherence to the diet via a secure email link from RedCAP.

A text message support program, designed by A/Prof Brennan, Dr Devlin and Dr Parr will be implemented to encourage participant adherence to their respective interventions. Text messages will be tailored to participant's name and stage of intervention and will include practical ideas and suggestions for preventing and managing barriers to adherence. Text messages will be delivered using a Web-based bulk text message platform. Participants will receive three texts per week in M4, two per week in M5, and one per week for the final months of the follow-up period, as individualized and decreasing frequency of messages is associated with better outcomes.

In weeks 30-32, participants will be asked to upload photographs and record daily energy intake using the EDD app. In week 32, participants will attend an in-clinic visit for ~15 min for assessment of weight, blood pressure, and to discuss any changes in diet, symptoms or medications they may have had in the preceding month.

Physical activity / sleep patterns: In order to monitor physical activity levels, participants will wear the Activpal activity monitor for the 2-weeks preceding each metabolic visit. The monitor is the size of a domino and will be worn continuously on the quadricep of one leg. The ActivPal records information regarding the frequency and duration of times spent sitting, standing and moving to estimate the wearer's physical activity duration, number of steps taken and sleep duration, as well as estimating total energy expenditure.

Continuous glucose monitoring: Interstitial glucose concentrations will be continuously measured using a subcutaneous glucose monitor (FreeStyle Libre Pro, Abbott). A small glucose-oxidase based electrochemical sensor is inserted under the skin on the back of the upper arm, and will remain *in situ* for 14 days. The electrode automatically records and stores glucose levels every 15 minutes. The data is stored by the sensor until the participant returns to the department, at which time the sensor is scanned and reports downloaded.

Analysis

Statistical analysis: The primary analysis is a linear regression of the primary endpoint, HbA1c assessed at 4 months. In addition to the treatment effect estimate, this analysis will adjust for baseline HbA1c, site and other factors prognostic for HbA1c. If the upper 95% confidence interval of the treatment effect estimate (TRE vs CP) is below 0.2% then non-inferiority will

be concluded. Subsequently if the upper 95% confidence interval is below 0% then superiority of TRE vs CP will be concluded. A secondary analysis of the primary endpoint will consist of extending this mixed effects regression to include HbA1c measures at 3 and 12 months and include assessment as a fixed effect and random intercepts per individual. All analyses will be performed according to the intent-to-treat principle with individuals analysed in the group they were allocated. With every effort made to ensure all randomized individuals are assessed at 4 months irrespective of compliance. A detailed analysis plan will be completed and uploaded onto the Clinical Trials register.

7. Confidentiality, data storage and security

All care will be taken to respect the privacy and confidentiality of participants in all interactions. Upon screening, participants will be allocated an ID code, and all materials de-identified by the investigator conducting the screening. Information collected during the study period will be identified with the study ID only. Electronic documents with personal information will be saved in a separate password-protected database with limited access by study researchers only. Hard copies of data and the signed consent form will be stored in a locked cabinet on level 7 at SAHMRI (Adelaide site) and in a filing cabinet in the clinical data storeroom on level 5 at MMIHR (Melbourne site). The master sheet containing identifiable data (name, email address) and ID code will be password protected and accessible only by the researchers named on this protocol. Personal information from study participants will not be disclosed to anyone unless required by law or upon personal consent.

De-identified information collected during the study period including demographical information (e.g. age, sex, ethnicity), anthropometric measurements (i.e. height, weight, waist/hip circumference), blood pressure, frozen blood samples (i.e. whole blood, serum, plasma, buffy coat); questionnaire data and the adverse event record may be accessed in the future by other researchers (i.e. future PhD students) and research placement students working under direct supervision of the named investigators. Plasma and serum samples will be stored for future analysis of markers of interest that are outside the scope of the current grant (i.e. inflammatory markers). A statement is included in the PIS asking participants to give or withhold their extended consent for use of biological samples and data in future research. Only data from participants who agree to give this 'extended' consent will be stored and/or shared for future research use.

Upon completion of the study, all study data will be digitized and stored on the password protected database, with the exception of consent forms; hard copies will be retained in a locked cabinet at SAHMRI. Biospecimens will be stored in A/Prof Heilbronn's -80 freezer on level 7 of SAHMRI and in Prof Hawley's -80 freezer on level 1 of the Daniel Mannix Building (Room 4.03.01) at ACU, for a minimum of 5 years after publication, up to a maximum of 15 years. They will be identified by study code only. All other records and materials will be retained for a minimum of 15 years from the date of publication. A/Prof Heilbronn will be responsible for ensuring all data is disposed of appropriately after this date.

8. Publication

Data collected from this study will be published in scientific journals and presented at academic conferences. No individual data will be reported; all outcomes will be reported as descriptive statistics (i.e. mean, median) of the cohort. Individual, de-identified data will be available on request.

9. Ethical considerations

Benefits of the study: This study is not directly assessing a treatment for a disease, and as such, participants will not directly benefit from participating. However, we will be able to offer participants information about how they responded to the treatment, compared with other volunteers. Some of the outcomes we measure (such as cholesterol and fasting blood glucose) are indicators of health and well-being. If these measures are outside of the normal ranges, participants will be notified and provided with the relevant information to take to their GP for further evaluation. Whilst not diagnostic, if answers provided on questionnaires (i.e. DASS) indicate that a participant may be in distress, or at-risk, they will be referred to their GP for follow up. At the end of the study, participants will be invited to return for an information evening / online webinar where we will tell participants about the outcomes of the research.

The outcomes of the study represent potential benefits to the community. This 12 month, multi-centre randomised clinical trial will be the first to determine whether TRE improves glycaemic control in individuals at risk of developing T2DM. If we determine TRE is an effective and acceptable dietary tool that improves glucose control and metabolic health, then in collaboration with the health service groups identified, TRE could be rapidly implemented in the primary clinical setting to a growing number of individuals at risk for developing T2DM. Results from this study will also provide a strong evidence base and further tools for the development of community-based interventions and public health advice on the health benefits of adopting TRE.

Risks and burdens of the study:

For participants:

The time to participate in the study is a total of 12.5 months. This will require participants to attend the clinical facility in their city (either Adelaide or Melbourne) on eight occasions (screening, 3 x metabolic visits, 1x check-in visit, 3x ~ 30 min visits to collect CGM/ActivPal) for a total of ~ 7 hours. They will also have 5x telehealth appointments with an APD, for a total of ~ 2.5 hours over the course of the study. To offset the burden of time spent in our clinics, travel and parking, participants will be offered an honorarium of $300, half of which will be paid at M4 and half on completion of the study. Recording of data using the food diary, and the wearing of the ActivPal activity monitor also represent a potential burden to the participants.

The metabolic testing visits are associated with several risks, including temporary discomfort during blood pressure measurements. Venepuncture for blood sampling may cause mild pain, light-headedness, bleeding, bruising and infection and can occasionally cause mild irritation or clotting of the vein. Placement of the CGM probe may cause slight discomfort at the site of insertion; this is generally transient and no anaesthetic is required. Additional, less common, risks include slight bruising, bleeding and local infection at the site. Generally, these effects are temporary and are expected to resolve completely. All risks and burdens are outlined to participants in the PIS and will be verbally explained to them at the screening visit. Participants will be given a printed copy of the PIS at the screening visit, to retain for their own records.

The DXA scan is associated with exposure to a very small amount of ionising radiation. The effective dose from this study is about 0.012 millisieverts (all participants will receive 3 DXA [0.004 millisieverts] scans to assess changes in body composition, at baseline and study end). At this dose level, no harmful effects of radiation have been demonstrated, as any effect is too small to measure. The risk is believed to be very low. A radiation safety report is attached for review (attachment 5: radiation safety reports).

Whilst unlikely, the standardised questionnaires and qualitative interviews may cause distress in some participants. A distress protocol has been established and is attached for review (attachment 6: distress protocol).

An independent Data Safety Management Committee (DSMC) will be established. Interim reports will be provided on ethics amendments, recruitment, protocol violations, and AEs at pre-specified completion intervals. Any serious adverse events likely to have occurred as a result of the study will be reported to the Chairman of the Ethics Committee within 72 hours. In the event of a data breach, the University of Adelaide's data breach response plan will be followed.

For researchers:

Handling of human bio-specimens presents a risk of potential blood-borne infections due to spills, or a needle stick injury. All staff collecting and handling bio-specimens will be appropriately trained, will be required to be up to date with vaccinations (i.e. Hepatitis A and B), and will wear appropriate PPE at all times, as outlined in standard operating procedures established at each site.

All procedures described are well established in our laboratories, all researchers are trained and experienced in the procedures, and SOP's are established. If a participant becomes distressed at any point during a procedure, the procedure will be discontinued immediately. Participants will be given the option to continue with the remainder of the study if they choose.

The project will be supported by research funds from a Medical Research Future Fund (MRFF) awarded to A/Prof Heilbronn. Researchers will be indemnified by their respective University.

10. References

[1] Diabetes Prevention Program Research G, Knowler WC, Fowler SE, et al. (2009) 10-year follow-up of diabetes incidence and weight loss in the Diabetes Prevention Program Outcomes Study. Lancet 374: 1677-1686

[2] Wing RR, Phelan S (2005) Long-term weight loss maintenance. The American Journal of Clinical Nutrition 82: 222S-225S

[3] Zimmet P, Alberti KGMM, Stern N, et al. (2019) The Circadian Syndrome: is the Metabolic Syndrome and much more! J Intern Med 286: 181-191

[4] Parr EB, Heilbronn LK, Hawley JA (2020) A Time to Eat and a Time to Exercise. Exercise and Sport Sciences Reviews 48: 4-10

[5] Sutton EF, Beyl R, Early KS, Cefalu WT, Ravussin E, Peterson CM (2018) Early Time-Restricted Feeding Improves Insulin Sensitivity, Blood Pressure, and Oxidative Stress Even without Weight Loss in Men with Prediabetes. Cell Metabolism 27: 1212-1221.e1213

[6] Kant AK, Graubard BI (2015) 40-year trends in meal and snack eating behaviors of American adults. Journal of the Academy of Nutrition and Dietetics 115: 50-63

[7] Riou J, Lefèvre T, Parizot I, Lhuissier A, Chauvin P (2015) Is There Still a French Eating Model? A Taxonomy of Eating Behaviors in Adults Living in the Paris Metropolitan Area in 2010. PLOS ONE 10: e0119161

[8] Timperio A, Worsley A, Livingstone KM, McNaughton SA, Leech RM (2017) Temporal eating patterns: associations with nutrient intakes, diet quality, and measures of adiposity. The American Journal of Clinical Nutrition 106: 1121-1130

[9] Gill S, Panda S (2015) A Smartphone App Reveals Erratic Diurnal Eating Patterns in Humans that Can Be Modulated for Health Benefits. Cell Metabolism 22: 789-798

[10] Zarrinpar A, Chaix A, Panda S (2016) Daily Eating Patterns and Their Impact on Health and Disease. Trends in Endocrinology & Metabolism 27: 69-83

[11] Hawley JA, Sassone-Corsi P, Zierath JR (2020) Chrono-nutrition for the prevention and treatment of obesity and type 2 diabetes: from mice to men. Diabetologia

[12] Parr EB DB, Radford BE, Hawley JA. (2020) Delaying breakfast as a modified time-restricted 2 feeding protocol for improving glycemic control and 3 encouraging dietary adherence for men with 4 overweight/obesity: a randomized controlled trial. Nutrients Accepted for Publication, Feb 3

[13] Hutchison AT, Regmi P, Manoogian E, Panda S, Wittert G, Heilbronn LK (2019) Time restricted feeding improves glucose metabolism in men at risk of type 2 diabetes. Obesity (Silver Spring) In Press

[14] Parker AR, Byham-Gray L, Denmark R, Winkle PJ (2014) The Effect of Medical Nutrition Therapy by a Registered Dietitian Nutritionist in Patients with Prediabetes Participating in a Randomized Controlled Clinical Research Trial. Journal of the Academy of Nutrition and Dietetics 114: 1739-1748

[15] Lindström J, Louheranta A, Mannelin M, et al. (2003) The Finnish Diabetes Prevention Study (DPS). Lifestyle intervention and 3-year results on diet and physical activity 26: 3230-3236

[16] Council. NHaMR (2013) National Health and Medical Research Council (2013) Australian Dietary Guidelines. Canberra:. In, https://www.nhmrc.gov.au/about-us/publications/australian-dietary-guidelines
[17] Richardson J, Iezzi A, Khan MA, Maxwell A (2014) Validity and reliability of the Assessment of Quality of Life (AQoL)-8D multi-attribute utility instrument. Patient 7: 85-96
[18] Lovibond SHL, P.F. (1995) Manual for the Depression Anxiety Stress Scales
[19] Fairburn CG, Beglin SJ (1994) Assessment of eating disorders: interview or self-report questionnaire? Int J Eat Disord 16: 363-370
[20] Bohn K, Doll HA, Cooper Z, O'Connor M, Palmer RL, Fairburn CG (2008) The measurement of impairment due to eating disorder psychopathology. Behav Res Ther 46: 1105-1110
[21] Buysse DJ, Reynolds CF, 3rd, Monk TH, Berman SR, Kupfer DJ (1989) The Pittsburgh Sleep Quality Index: a new instrument for psychiatric practice and research. Psychiatry Res 28: 193-213
[22] Horne JA, Ostberg O (1976) A self-assessment questionnaire to determine morningness-eveningness in human circadian rhythms. Int J Chronobiol 4: 97-110

Summary of amendments approved

Date:	Amendments to protocol:
December 2020	• Addition of new investigators to protocol. Staff names added to the Protocol, Participation Information Sheet and consent form.<br>• Clarification of statistical analysis plan and non-inferiority margin.<br>• Amendment of diet description in PIS to mask whether participants are randomised to control (current practice) or intervention (TRE), which also necessitated change in study title.<br>• Addition of secondary outcomes to the protocol and PIS.<br>• Change final eating time from 7:30 pm to 7:00 pm
March 2021	• Change Easy Diet Diary to Research Food Diary.<br>• Addition of quantification of diet quality by HEIFA<br>• Remove criteria around alcohol intake<br>• Include Nutrition Quality Of Life questionnaire<br>• Wording changed around dietitian consults (PIS) and Protocol to clarify that the TRE group will only be receiving advice around changing when they eat – no "dietetics" information.<br>• Nomenclature of visits changed (screening is now V-2)<br>• Study title updated on consent form (typo from prev version) (Now version 2)<br>• 2 week diet recording in week 30 added to PIS –omitted (typo) in previous version but was included in protocol
May 2021	• Include risks associated with Ambulatory blood pressure monitoring<br>• Add in Personality inventory for DSM-5
August 2021	• Addition of two new staff members. Staff names added to the Protocol, Participation Information Sheet and consent form.
June 2022	• Addition of four staff members- Staff names added to the Protocol and Participation Information Sheet.<br>• Principal Investigator title changed from Associate Professor to Professor on Protocol, PIS and Consent.<br>• Oversight of past title change from consent form - title of Consent form changed from previous title "Treat" to current title "What Or When to eat to reduce the risk of type 2 diabetes (WOW study)".
August 2022	• Addition of one staff member- Staff name added to the Protocol, Participation Information Sheet and consent form.<br>• Addition of recruitment via radio advertisement

February 2023	• Addition of one staff member. Staff name added to the Protocol, Participation Information Sheet and consent form.
July 2023	• Addition of two staff members- Staff Names added to the Protocol and Participation Information Sheet.<br>• Inclusion of final statistical analysis plan

Electronic Supplementary Material

ESM 2

Electronic supplementary material methods

Self-reported adherence

Participants were asked to complete ratings of overall adherence to their randomised dietary intervention, between 1 (never), 2 (1-2 days per week), 3 (3-4 days per week), 4 (5-6 days per week) or 5 (everyday), at 9 timepoints (0.5, 1, 2, 3, 3.5, 4, 8, 11.5 and 12 months). In analysis, these responses were converted into numbers: 0, 1.5, 3.5, 5.5 or 7. These scores were then averaged over the period, either 0-4 mo or 4-12 mo, weighted by the time duration between responses. When a score was missing the scoring was weighted by the frequency and distribution of responses.

Electronic supplementary material tables

Fidelity analysis

ESM Table 1. Consult attendance between groups

	**TRE**	**IDG[1]**
**Number of consults attended (attended at least one consult), n (%)**		
Five	108 (89%)	107 (88%)
Four	8 (7%)	8 (7%)
Three	2 (2%)	4 (3%)
Two	3 (2%)	2 (2%)
One	3 (2%)	1 (1%)
**Number of consults attended (completers), n (%)**		
Five	106 (94%)	106 (95%)
Four	6 (5%)	5 (4%)
Three	1 (<1%)	1 (<1%)
Two	0	0
One	0	0

[1] One participant randomised to IDG discontinued the study after randomisation and before completing the first diet consult.

Protocol violations

ESM Table 2. Summary of protocol violations.

Group	Reason	Detail
TRE	Exclusion: Eating Disorder Examination ≥2.8	EDEQ score = 2.9
IDG	Exclusion: Eating Disorder Examination ≥2.8	EDEQ score = 2.8
IDG	Exclusion: Eating Disorder Examination ≥2.8	EDEQ score = 2.8
IDG	Inclusion: Aged 35 to 70 years	Age = 34 years
IDG	Inclusion: Overweight or obese (BMI: ≥25 but <45 kg/m^2)	BMI = 45 kg/m^2

ESM Table 3. Changes in absolute macronutrient intake after the 4 months intervention and after 12 months.

	n		**Estimated change from baseline (95% CI)**		**Between group difference (95% CI)**	**Between group p-value**
	**TRE**	**IDG**	**TRE**	**IDG**	**TRE vs IDG**	
**Dietary intake**						
Carbohydrate (g)						
Month 4	82	95	-10.3 [-21.4, 0.9]	**-45.2 [-54.9, -35.6]**	35.0 [21.9, 48.1]	<0.0001
Month 12	70	77	**-19.8 [-34.3, -5.3]**	**-46.2 [-59.1, -33.3]**	26.4 [8.8, 44.0]	0.003
Total sugar (g)						
Month 4	82	95	**-6.1 [-12.0, -0.3]**	**-17.2 [-22.3, -12.1]**	11.1 [4.1, 18.0, 4.1]	0.002
Month 12	70	77	**-14.4 [-22.0, -6.9]**	**-21.7 [-28.4, -15.1]**	7.7 [-1.0, 16.4]	0.11
Total fat (g)						
Month 4	82	95	**-5.6 [-10.7, -0.6]**	**-19.2 [-23.6, -14.8]**	13.6 [7.6, 19.6]	<0.0001
Month 12	70	77	**-7.7 [-14.1, -1.4]**	**-19.2 [-24.9, -13.5]**	11.5 [3.7, 19.2]	0.004
Saturated fat (g)						
Month 4	82	95	**-2.4 [-4.5, -0.3]**	**-8.9 [-10.7, -7.0]**	6.5 [4.0, 9.0]	<0.0001
Month 12	70	77	-2.7 [-5.5, 0.1]	**-7.1 [-9.5, -4.6]**	4.0 [0.6, 7.3]	0.01
Protein (g)						
Month 4	82	95	**-5.9 [-11.3, -0.4]**	**-6.6 [-11.3, -1.8]**	0.7 [-5.8, 7.2]	0.83
Month 12	70	77	**-6.7 [-12.7, -0.7]**	**-12.3 [-17.7, -6.9]**	5.6 [-1.8, 13.0]	0.13
Fibre (g)						
Month 4	82	95	-1.4 [-3.0, 0.3]	-1.9 [-3.4, -0.5]	0.6 [-1.4, 2.5]	0.58
Month 12	70	77	**-1.6 [-3.6, 0.4]**	**-2.5 [-4.3, -0.7]**	0.9 [-1.5, 3.3]	0.45
Alcohol (g)						
Month 4	82	95	-2 [-4, 0]	-1 [-3, 1]	-1 [-3, 2]	0.66
Month 12	70	77	-2 [-5, 0]	**-4 [-6, -1]**	2 [-2, 5]	0.31

Data are presented as change from baseline and mean difference between groups using means and 95% confidence intervals. Exploratory outcomes were analysed using linear regressions adjusting for baseline HbA1c (continuous), site (Adelaide or Melbourne) and sex (male or female). These analyses were complete case analyses. Dietary intake was calculated from objective data collected from Research Food Diary (5 days). IDG individualized dietetic guidance group; TRE time-restricted eating group.

Sensitivity analysis

ESM Table 4. Sensitivity analyses of the primary outcome, HbA1c assessed at 4 months.

	**Estimated change from baseline (95% CI)**		**Mean difference between groups (95% CI)**	**Between group p-value**
	**TRE**	**IDG**	**TRE vs IDG**	
**Complete Case**				
HbA1c (%)				
Month 4	-0.03 [-0.07, 0.006]	-0.02 [-0.06, 0.02]	-0.01 [-0.06, 0.03]	0.51
Month 12	-0.0006 [-0.05, 0.05]	-0.05 [-0.1, 0.002]	0.05 [-0.008, 0.1]	0.09
**Worst Case (imputed)**				
HbA1c (%)				
Month 4	-0.04 [-0.08, 0.004]	-0.02 [-0.06, 0.03]	-0.02 [-0.07, 0.03]	0.41
Month 12	0.009 [-0.05, 0.06]	-0.03 [-0.09, 0.02]	0.04 [-0.11, 0.02]	0.17

In general, to convert HbA1c from percentage to mmol/mol, use the following formula: HbA1c (mmol/mol) = 10.929 × (HbA1c [%] −2.15). Therefore, for the difference between groups ΔHbA1c (mmol/mol) = 10.929 × ΔHbA1c (%).

ESM Table 5. Other exploratory outcomes.

	n		Estimated change from baseline (95% CI)		Mean difference between groups (95% CI)	Between group p-value
	TRE	IDG	TRE	IDG	TRE vs IDG	
**CGM metrics**						
Time below range (%)	106	100	0.8 [-2.0, 3.5]	1.9 [-0.8, 14.7]	-1.2 [-4.7, 2.3]	0.50
Time in range (%)	106	100	-1.0 [-3.7, 1.7]	-2.4 [-0.3, -5.1]	1.4 [-2.0, 4.9]	0.40
Time above range (%)	106	100	0.06 [-0.7, 0.7]	0.3 [-0.4, 1.0]	-0.2 [-1.1, 0.7]	0.59
iAUC (24-h)	106	100	-0.41 [-0.97, 0.15]	-0.24 [-0.79, 0.32]	-0.18 [-0.89, 0.54]	0.62
SD	106	100	-0.003 [-0.006, 0.0001]	-0.002 [-0.005, 0.001]	-0.0009 [-0.005, 0.003]	0.66
Mean wear time (days)*	106	100	12.3 ± 2.0	12.0 ± 2.0	--	--
**Liver markers**						
ALT (U/L)†						
Month 4	114	111	-0.05 [-0.11, 0.007]	-0.02 [-0.08, 0.04]	-0.03 [-0.11, 0.04]	0.41
Month 12	96	95	-0.04 [-0.1, 0.04]	-0.05 [-0.1, 0.03]	0.009 [-0.1, 0.1]	0.86
AST (U/L)†						
Month 4	114	111	**-0.04 [-0.08, -0.002]**	0.003 [-0.04, 0.04]	-0.04 [-0.09, 0.005]	0.08
Month 12	96	95	-0.02 [-0.1, 0.03]	-0.008 [-0.1, 0.04]	0.02 [-0.1, 0.05]	0.62
**Body composition**						
Waist to hip ratio						
Month 4	112	111	-0.003 [-0.01, 0.006]	0.002 [-0.006, 0.01]	-0.005 [-0.02, 0.005]	0.31
Month 12	95	95	0.009 [-0.0006, 0.02]	**0.01 [0.0005, 0.02]**	-0.0008 [-0.01, 0.01]	0.88

Data are represented as change from baseline and mean difference between groups using means and 95% confidence intervals, presented to the nearest whole number). Exploratory outcomes were analysed using linear regressions adjusting for baseline HbA1c (continuous), site (Adelaide or Melbourne) and sex (male or female). These analyses were complete case analyses. Bold indicates significant ($p<0.05$) within group change. *indicates data from 4-months, where minimum wear time was 1.0 days, baseline data is in Table 1; †indicates log transformed data.

Adverse events

ESM Table 6. Adverse events reported by participants during the 12-month intervention and follow up periods.

AE Classification	Max Grade[1]	DIET n=123	TRE n=124	All n=247	p-value[2]
Allergic reaction	3	1 (<1%)	0 (0%)	1 (<1%)	
Anxiety/Depression	1	0 (0%)	3 (2%)	3 (1%)	
Arthritis	1	0 (0%)	1 (<1%)	1 (<1%)	
	2	1 (<1%)	0 (0%)	1 (<1%)	
Bruising	1	0 (0%)	2 (2%)	2 (<1%)	
Bullous dermatitis	2	1 (<1%)	0 (0%)	1 (<1%)	
Burn	2	0 (0%)	1 (<1%)	1 (<1%)	
Cancer - prostate	3	0 (0%)	1 (<1%)	1 (<1%)	
Colitis	1	0 (0%)	1 (<1%)	1 (<1%)	
	2	0 (0%)	1 (<1%)	1 (<1%)	
	3	0 (0%)	1 (<1%)	1 (<1%)	
Constipation	1	1 (<1%)	2 (2%)	3 (1%)	
Diarrhea	1	8 (6%)	2 (2%)	10 (4%)	0.60
	2	1 (<1%)	4 (3%)	5 (2%)	
Dyspepsia	1	1 (<1%)	1 (<1%)	2 (<1%)	
Dyspnea	1	1 (<1%)	0 (0%)	1 (<1%)	
	2	0 (0%)	1 (<1%)	1 (<1%)	
Fall	1	1 (<1%)	0 (0%)	1 (<1%)	1.00
	2	1 (<1%)	2 (2%)	3 (1%)	
	3	1 (<1%)	1 (<1%)	2 (<1%)	
Fatigue	1	4 (3%)	6 (5%)	10 (4%)	0.54
Flu like symptoms	1	32 (26%)	24 (20%)	56 (23%)	0.31
	2	37 (30%)	38 (31%)	75 (30%)	
	3	2 (2%)	0 (0%)	2 (<1%)	
Fracture	1	2 (2%)	1 (<1%)	3 (1%)	
Gallbladder obstruction	3	0 (0%)	1 (<1%)	1 (<1%)	
Gastroesophageal reflux	1	0 (0%)	2 (2%)	2 (<1%)	
Gastrointestinal disorder	2	1 (<1%)	2 (2%)	3 (1%)	
Hand-Foot Syndrome	2	0 (0%)	1 (<1%)	1 (<1%)	
Headache	1	3 (2%)	4 (3%)	7 (3%)	1.00
	2	2 (2%)	0 (0%)	2 (<1%)	
Heart failure	1	0 (0%)	1 (<1%)	1 (<1%)	
Hypertension	2	1 (<1%)	0 (0%)	1 (<1%)	
Infection	1	3 (2%)	6 (5%)	9 (4%)	0.24
	2	7 (6%)	9 (7%)	16 (6%)	
	3	1 (<1%)	2 (2%)	3 (1%)	
Inflammation Middle Ear	1	0 (0%)	1 (<1%)	1 (<1%)	
	2	0 (0%)	1 (<1%)	1 (<1%)	
Irritability	1	0 (0%)	1 (<1%)	1 (<1%)	
Laryngitis	1	1 (<1%)	1 (<1%)	2 (<1%)	
Lymphedema	1	0 (0%)	1 (<1%)	1 (<1%)	

Menorrhagia	2	1 (<1%)	0 (0%)	1 (<1%)	
Nausea/Vomiting	1	2 (2%)	2 (2%)	4 (2%)	0.68
	2	0 (0%)	1 (<1%)	1 (<1%)	
Neoplasms	3	2 (2%)	0 (0%)	2 (<1%)	
Osteoarthritis	2	0 (0%)	1 (<1%)	1 (<1%)	
Pain	1	4 (3%)	4 (3%)	8 (3%)	0.36
	2	4 (3%)	8 (7%)	12 (5%)	
Palpitations	1	1 (<1%)	0 (0%)	1 (<1%)	
Pancreatitis	4	1 (<1%)	0 (0%)	1 (<1%)	
Periodontal disease	1	0 (0%)	3 (2%)	3 (1%)	
Presyncope	1	4 (3%)	2 (2%)	6 (2%)	1.00
	2	0 (0%)	1 (<1%)	1 (<1%)	
Psychiatric disorders	1	0 (0%)	1 (<1%)	1 (<1%)	
	2	0 (0%)	1 (<1%)	1 (<1%)	
Shingles	2	1 (<1%)	0 (0%)	1 (<1%)	
Sinus disorder	1	0 (0%)	1 (<1%)	1 (<1%)	
Stroke	3	1 (<1%)	0 (0%)	1 (<1%)	
Urostomy stenosis	3	0 (0%)	1 (<1%)	1 (<1%)	

[1]If an individual experienced the same adverse event on multiple occasions, only the event with the maximum grade was reported. [2]When there were at least four individuals who experienced a particular adverse event, a Fisher exact test was conducted comparing the rates of occurrence (irrespective of grade) between the two intervention groups. No adjustments for multiple testing were performed.

Electronic supplementary material Figure

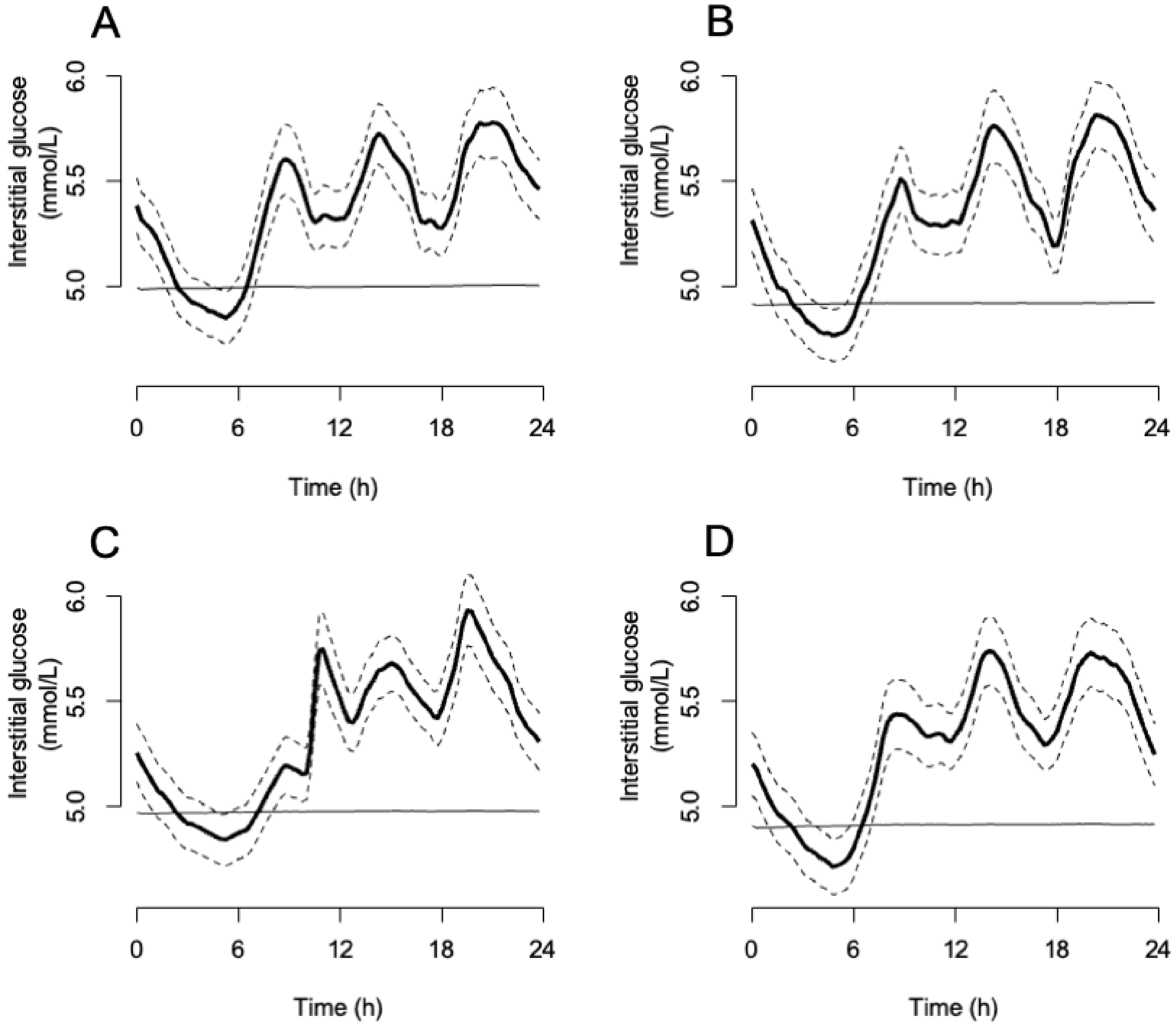

ESM Figure 1. 24-h mean and 95% CI interstitial glucose profiles during baseline periods for the (A) TRE group and (B) IDG group, and 4-month follow ups for (C) TRE and (D) IDG. The black line represents the mean data, the dotted lines representing the 95% confidence bands, and the straight line represents the mean daily CGM baseline as assessed using the 40th percentile method described by Chkroun *et al* [29].

ESM 3

WOW: STATISTICAL ANALYSIS PLAN

Study Name: What Or When to eat to reduce the risk of type 2 diabetes (WOW)

Trial registration number: NCT04762251

SAP Author: Andrew Vincent

SAP Date: 29/02/2024

Version: 1

Signatures

Andrew Vincent ______ 29/2/24 ______

Leonie Heilbronn ______ 29/2/24 ______

John Hawley ______ 01/03/2024

CONTENTS

Abbreviations (page 3)

Preface (page 4)

Study Objectives (page 5)
- Primary
- Secondary
- Exploratory

Study Outcomes (page 6)
- Primary outcomes
- Secondary outcomes
- Exploratory outcomes

Study Details (page 7)
- Study design
- Schedule of assessments
- Overview of data assessments
- Study Population: Inclusion/exclusion criteria
- Sample size considerations
- Randomization details

Estimand Considerations (page 10)
- Population
- Variable
- Treatments
- Population level summary
- Intercurrent events
- Intercurrent event strategies
- Sensitivity analysis

Statistical Considerations (page 11)
- Error control
- Analysis sets
- Covariate adjustment
- Descriptive statistics

Statistical Methods (page 12)
- Primary outcome
- Secondary outcomes
- Exploratory outcomes
- Safety outcomes

References (page 13)

Appendix: Imputation overview and details (page 14)

ABBREVIATIONS

AUC	Area under the curve
AUSDRISK	The Australian type 2 diabetes risk assessment tool
BMI	Body mass index
CGM	Continuous glucose monitoring
CP	Current best practice
DSMC	Data safety monitoring committee
HbA1c	Glycated haemoglobin
iAUC	Incremental area under the curve
M0, M4, M12	M0 = baseline; M4 = 4 month; and M12 = 12 month assessments
SAP	Statistical analysis plan
TRE	Time restricted eating
T2DM	Type II diabetes mellitus
WOW	Study: What Or When to eat to reduce the risk of type 2 diabetes

PREFACE

This statistical analysis plan (SAP) describes the planned analyses and reporting for the WOW study to compare the impact of time restricted eating (TRE) versus current best practice (CP) in adults at high risk of 2 diabetes. The purpose of this SAP is to outline the considerations and the pre-specified analyses for the WOW study.

The project is funded by the Medical Research Future Fund Preventative and Public Health Grant MRF1200555 awarded to Prof Heilbronn.

This study has been approved by the Central Adelaide Local Health Network Human Research Ethics Committee (#14023).

Study Objectives

Primary

To determine whether TRE is not inferior to current practice guidelines (CP) in dietetics to reduce HbA1c at 4 months, in individuals at high risk of developing T2DM.

Secondary

Secondary aim of this study is to assess the short (4 month) and long term (12 month) effect of TRE on HbA1c and glucose metabolism.

Exploratory

The exploratory aims of this study are to assess:

- Adherence of TRE vs CP over 4 and 12 months.
- The impact of TRE vs CP at 4 and 12 months on cardio-metabolic health.
- The impact of TRE vs CP at 4 months on 24-h profiles of glycaemia by CGM

Study Outcomes

Primary objective: 4 months

Primary efficacy outcome: Change in glycated haemoglobin (HbA1c)

Secondary efficacy outcomes

- Change in fasting glucose, insulin, and HOMA-IR
- Nocturnal glucose CGM AUC (midnight to 4 am)

Secondary objective: 12 months

Secondary efficacy outcomes

- Change in HbA1c
- Change in fasting glucose concentrations, insulin concentrations, HOMA-IR

Exploratory objective: Adherence

- Adherence: Self-reported (Assessed: Month 0.5, 1, 2, 3, 3.5, 4, 8, 11.5, 12)
- Diet records (energy, macronutrient intake) by diary (Assessed M0, M4, M12)
- Meal timing / eating window by self-reported meal timing, time stamped food photos (Assessed M0, M4, M12)

Exploratory objective: Cardiometabolic

- Cardiometabolic outcomes: C-reactive protein concentrations, blood lipid concentrations, blood pressure, heart rate (Assessed: M0, M4, M12)
- Liver health outcomes: Change in ALT, AST (Assessed: M0, M4, M12)
- Body mass and body composition by DXA, Waist and hip circumferences (Assessed: M0, M4, M12).
- Physical activity and sleep by inclinometer (ActivPAL), sleep assessed by questionnaire (Assessed: M0, M4, M12)
- Chronotype: by MEQ-SA (Assessed M0, M4, M12)

Exploratory objective: CGM measures

1) Change in 24-h profiles of glycaemia (i.e., iAUC, time-in range, glucose variability) by CGM (FreeStyle Libre Pro), (Assessed: M0, M4)

(Visit notation: M0 = baseline; M4 = 4 months; and M12 = 12 months.)

Study Details

Study Design: WOW is a parallel, single-blinded, multi-centre randomised controlled clinical trial.

Figure 1: Schedule of assessments

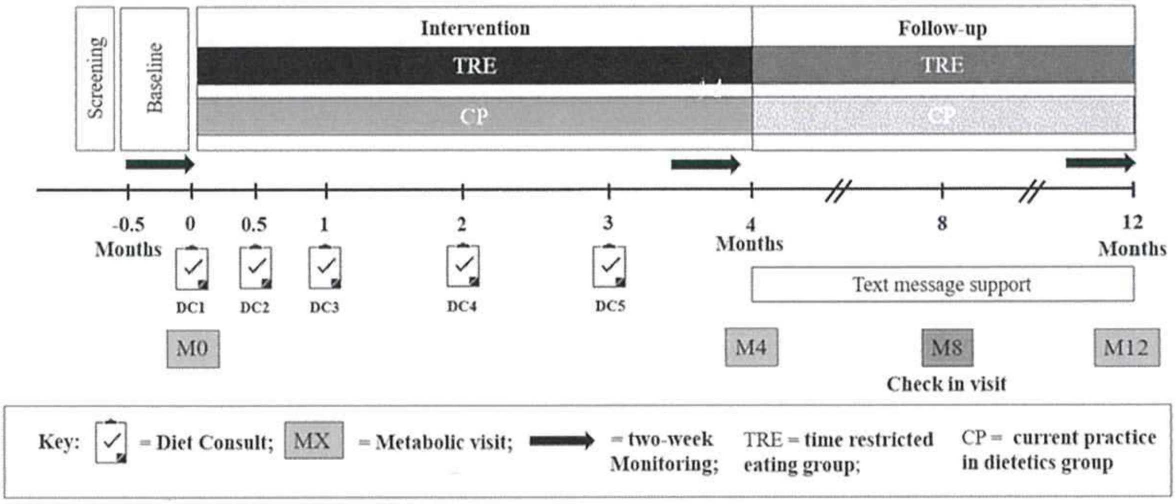

Study Population

Inclusion criteria

Study participants will be aged 35 to 70 years, overweight or obese (BMI: ≥25 but <45 kg/m^2) and will score ≥15 on the AUSDRISK assessment tool.

Exclusion criteria

- Type 1 or type 2 diabetes, or diabetes detected at screening HbA1c ≥6.5% (48 mmol/mol).
- The following medical conditions: Major psychiatric disorders (schizophrenia, major depressive disorder, bipolar disorder, eating disorders)
- Gastrointestinal disorders/disease (including malabsorption)
- Haematological disorders (i.e. thalassemia, iron-deficiency anaemia)
- Insomnia
- Currently receiving, or have received treatment/diagnosis of cancer in the past 3 years (excluding non-melanoma skin cancer)
- Significant liver or kidney disease requiring ongoing medical care
- Previous or planned gastro-intestinal surgery (including bariatric surgery)
- Congestive heart failure (NYHA stage 2 or above)
- Previous myocardial infarction or significant cardiac event ≤ 6 months prior to screening
- Previous cerebrovascular event ≤12 months prior to screening, and/or any other condition deemed unstable by the study physician.
- Any medication used, or known to lower blood glucose, or antidiabetic medications; diuretics or combination blood pressure medications containing a diuretic; beta-blockers; glucocorticoids; anti-epileptic medications; antipsychotic medications; Medications affecting weight, appetite or gut motility, opioid medications unless combined with paracetamol in a single formulation and used occasionally on a PRN basis
- Night shift work or individuals who work any shifts after 10 pm.
- Do not consume a regular breakfast (i.e. do not eat breakfast on an average of 5 or more days per week), and do not eat for more than 12 hours per day on an average of 5 or more days per week*
- Have an extreme or restricted pattern of eating (i.e. following an intermittent fasting diet) or already engaged in a TRE protocol
- Pregnant, planning a pregnancy or currently breastfeeding
- Those who have lost or gained >5% of body weight in the last 6 months
- Current smokers of cigarettes/marijuana/e-cigarettes/vaporisers
- Anyone unable to comprehend the study protocol or provide informed consent (i.e. due to English language or cognitive difficulties)

- Participants will not have seen a dietitian in the preceding 3 months.
- Kessler Psychological Distress Scale ≥30
- Eating Disorder Examination Questionnaire ≥2.8

Sample size considerations

In a population of individuals with pre-diabetes, the difference of 0.2% in mean HbA1c was shown to be clinically relevant (Lindström 2003). As such we take the non-inferiority margin to be 0.1% and power our study for an additional benefit of 0.1% of TRE over control (i.e. powered for 0.2% mean difference). Literature suggests that the within group standard deviation to be 0.6% in this population of people with pre-diabetes (Parker 2014; Lindström 2003). Then with n=214 individuals with 4 months assessments (randomized 1:1 to each intervention), there is 80% power to conclude that TRE is non-inferior to CP with a margin of 0.1% in a baseline adjusted ANCOVA when the true difference is 0.1% in favour of TRE (2-sided alpha=0.05).

In planning the study we expected attrition at four months would be <20% requiring a sample size of 268. However due to slow accrual due to COVID and better than expected attrition (close to 10%) the target of n=214 was likely to be attained with n=247 randomized. Approval for this change has been obtained by the independent DSMC.

Randomization details

Stratified random length blocked randomisation by site (Adelaide or Melbourne) and baseline HbA1c (<5.7% vs ≥5.7 to 6.5%)

Estimand Considerations

Population

- Australian adults with obesity and at risk of diabetes (see inclusion/exclusion criteria above)

Variable

- Change from baseline in glycated haemoglobin (HbA1c) at 4 months.

Treatments

- Time restricted eating (TRE)
- Current best practice (CP)

Population Level Summary

Mean difference in HbA1c between treatment groups.

Intercurrent Events

- Discontinuation of diet for any reason (including: lifestyle, dislike of diet and non-related medical adverse events).
- Unable to attend four-month HbA1c assessment due to reasons unrelated to diet (including moving overseas or interstate).
- Drop-out of study with reason unspecified.

Intercurrent Event Strategies

- Due to the intervention being dietary modification it is assumed that efficacy outcomes are strongly tied to compliance, thereby intercurrent event strategies are defined as whether individuals were able to continue the diet or not.
- Treatment policy will be assumed for individuals with diet discontinuation by assuming zero compliance thereafter.
- Hypothetical policy will be assumed for individuals who left the study (without diet discontinuation) by imputing compliance as if they had continued with the study.
- For individuals with unspecified drop-out reason diet discontinuation (yes/no) will be imputed within treatment group, then compliance will be imputed accordingly (ie treatment or hypothetical policy).

Sensitivity Analysis: Worst case

- All individuals in the TRE group with drop-out reason unknown will be assumed that they had discontinued the diet.
- All individuals in the CP group with drop-out reason unknown will be imputed as if they had continued the diet.

Sensitivity Analysis: Complete case

- No imputation, the analysis will only include individuals with HbA1c assessed at 4 months. This analysis assumes the missing data are missing completely at random.

Statistical Considerations

Error control: For the primary outcome the difference between TRE and CP will be assessed for non-inferiority. If the non-inferiority null is rejected then superiority will be tested. Two-sided 95% confidence intervals will be reported (ie one-sided 2.5% type I error rate). No multiple test adjustments will be made for secondary and exploratory outcomes.

Analysis Sets: Analyses of efficacy outcomes at 4 and 12 months (HbA1c, fasting glucose, insulin, HOMA-IR) will include all randomized individuals via multiple imputation. This process also requires physical activity and compliance data, as such analyses of these outcomes will also include all randomized individuals. Analyses of all other (exploratory) outcomes will be complete case analyses.

Covariate adjustment: All comparisons of treatment effect will include adjustment for stratification factors (site and baseline HbA1c as a continuous variable) and for sex as it is known that men are higher risk for diabetes.

Descriptive Statistics: A CONSORT flow diagram will present the number of individuals who participated in online screening, and clinic visits 0-3 (Figure 1). Descriptive summary statistics will be reported for baseline characteristics of all individuals who were randomized.

Statistical Methods

Primary Outcome

The primary analysis of the primary outcome is a covariate adjusted linear regression of differences in HbA1c at 4 months, adjusting treatment group (TRE vs CP), baseline HbA1c (continuous), site (Adelaide vs Melbourne) and sex (male vs female).

Non-inferiority will be concluded if upper 95% confidence interval of the effect estimate of the difference between groups (TRE – CP) is less than 0.1%. If non-inferiority is concluded then superiority may also be concluded if the upper 95% confidence interval excludes a between group difference of 0%.

This analysis will be performed in all randomized individuals with individuals using multiple imputation using chained equations within treatment groups and combined using Rubin's rules. The variables used in the multiple imputation are HbA1c (M0, M4 & M12), age, sex, site, fasting glucose (M0, M4 & M12) and insulin (M0, M4 & M12) concentrations, steps per day (M0, M4 & M12), nocturnal glucose CGM AUC (M0, M4), reason for dropout (related/unrelated to diet) and average compliance (first 4 months, and 4 to 12 months). Compliance will be imputed as per the intercurrent event strategies and the average compliance calculated over each period. Imputation will use multiple chained equations (*mice* R package), details of which are presented in the Appendix.

Secondary & Exploratory Outcomes

Treatment effect for the change in HbA1c at 12 months and other outcomes required for the multiple imputation of the primary outcome (assessed at 4 and/or 12 months) will be assessed using the same methodology as for the primary outcome, i.e. generalized linear regressions with multiple imputation for missing data.

Other outcomes will be analysed using generalized linear regressions adjusting for the same covariates, however these analyses will be complete case analyses.

Safety Outcomes

Summaries, for all adverse events and for those events believed to be related to the intervention, will be reported. In both cases, if an individual has experienced the adverse event on multiple occasions, only the event with the maximum grade will be reported.

When there are at least four individuals who experience a particular adverse event, a Fisher exact test will be conducted comparing the rates of occurrence (irrespective of grade) between the two intervention groups. No adjustments for multiple testing will be performed.

REFERENCES

Parker AR, Byham-Gray L, Denmark R, Winkle PJ (2014) The Effect of Medical Nutrition Therapy by a Registered Dietitian Nutritionist in Patients with Prediabetes Participating in a Randomized Controlled Clinical Research Trial. Journal of the Academy of Nutrition and Dietetics 114: 1739-1748

Lindström J, Louheranta A, Mannelin M, et al. (2003) The Finnish Diabetes Prevention Study (DPS). Lifestyle intervention and 3-year results on diet and physical activity 26: 3230-3236

Appendix

Data pre-processing

CGM baseline glucose algorithm estimated using the algorithm provided below. Nocturnal CGM iAUC calculated as the area under the curve of CGM glucose minus baseline between 12 midnight and 4am.

Total number of steps is set to missing if the number of assessment days is less than 5.

Imputation Overview

Missing data is imputed within allocated treatment group.

HbA1c, fasting glucose concentration, fasting insulin concentration and steps-per-day are imputed on log scale and back transformed for analyses on the original scale.

Log of the total steps-per-day is analysed with an offset of log of number of days assessed.

Baseline variables are used for imputing all variables.

All variables within an assessment period are used per assessment period, diet-discontinue (no vs yes), average compliance (per period) and repeated measures of each variable are also included.

For example imputing 4 month (log) HbA1c uses all baseline variables, all 4 month variables, the binary diet-discontinue variable, the four month average compliance and the 12 month (log) HbA1c variable.

All variables are imputed with predictive mean matching except the compliance and diet-discontinue variables for which ordinal and logistic regressions are used respectively.

Imputation Code

```
####################
## Multiple Imputation
####################

set.seed(1234)

MNAR.type <- 0
## MNAR.type <- 0 => Primary estimand strategy
## MNAR.type <- 1 => Worst-case sensitivity analysis

## Imputation variables
{
  m0.list <- c(
    'state', 'age', 'sex'
    , "hba1c.log.v1", "glu.log.v1", "insulin.log.v1"
    , 'total.num.steps.log.v1', "cgm.iauc.v1"
  )
  m0.list <- m0.list[order(colSums(is.na(full.data[,m0.list])))]
  m2.list <- c('ice.grp', paste('m', c(0.5, 1, 2, 3, 3.5, 4), sep=''), "m4.compliance")
  m4.list <- c(
    "hba1c.log.v2", "glu.log.v2", "insulin.log.v2"
    , "total.num.steps.log.v2", "cgm.iauc.v2"
  )
  m4.list <- m4.list[order(colSums(is.na(full.data[,m4.list])))]
  m8.list <- c(paste('m', c(8, 12), sep=''), "m12.compliance")
  m12.list <- c("hba1c.log.v3", "glu.log.v3", "insulin.log.v3", "total.num.steps.log.v3")
  m12.list <- m12.list[order(colSums(is.na(full.data[,m12.list])))]
  aux.list <- c("id", "group", "drop.out.time", "num.valid.days.log.v1", "num.valid.days.log.v2", "num.valid.days.log.v3")
  table(table(c(m0.list, m2.list, m4.list, aux.list))) # all 1s

  var.list <- c(m0.list, m2.list, m4.list, m8.list, m12.list)
  #c(var.list, aux.list)[!c(var.list, aux.list) %in% names(full.data)]
  imp.data <- full.data[, c(var.list, aux.list)]
  colSums(is.na(imp.data))
}

## Sensitivity Analysis: Worse case
{
  if (MNAR.type == 1) {
    imp.data$ice.grp[imp.data$group. == 0 & is.na(imp.data$ice.grp)] <- 'Completer'
    imp.data$ice.grp[imp.data$group. == 1 & is.na(imp.data$ice.grp)] <- 'Discontinue diet'
  }
}
```

```
## Imputation formula per variable
{
  fm.list <- NULL
  for (var. in m0.list) {
    # var. <- m0.list[4]
    fm. <- paste(var., "~", paste(m0.list[!m0.list %in% c(var., 'total.num.steps.log.v1')], collapse=' + '))
    if (!grepl('total.num.steps.log', var.)) fm. <- paste(fm., ' + I(total.num.steps.log.v1 - num.valid.days.log.v1)')
    if (grepl('total.num.steps.log', var.)) fm. <- paste(fm., ' + offset(num.valid.days.log.v1) + I(total.num.steps.log.v2 - num.valid.days.log.v2) + I(total.num.steps.log.v3 - num.valid.days.log.v3)')
    if (var. %in% c("hba1c.log.v1", "glu.log.v1", "insulin.log.v1")) fm. <- paste(c(fm., sub('v1', 'v2', var.), sub('v1', 'v3', var.)), collapse=' + ')
    if (var. %in% c("cgm.iauc.v1")) {
      fm. <- paste(c(fm., sub("v1", "v2", var.)), collapse=' + ')
    }; fm.list <- c(fm.list, as.formula(fm.)); rm(fm.)
  }; rm(var.)
  for (var. in m2.list) {
    # var. <- m2.list[1]
    fm. <- paste(var., "~ (", paste(c(
      m0.list[!m0.list %in% c('total.num.steps.log.v1')], c('ice.grp', 'm4.compliance')[!c('ice.grp', 'm4.compliance') %in% var.]
      , m4.list[!m4.list %in% c('total.num.steps.log.v2')]
    ), collapse=' + '), ')')
    fm. <- paste(fm., ' + I(total.num.steps.log.v1 - num.valid.days.log.v1) + I(total.num.steps.log.v2 - num.valid.days.log.v2)')
    fm.list <- c(fm.list, as.formula(fm.)); rm(fm.)
  }; rm(var.)
  for (var. in m4.list) {
    fm. <- paste(var., "~ (", paste(c(
      m0.list[!m0.list %in% c('total.num.steps.log.v1')]
      , c('m4.compliance')
      , m4.list[!m4.list %in% c(var., 'total.num.steps.log.v2')]
    ), collapse=' + '), ')')
    fm. <- paste(fm., ' + I(total.num.steps.log.v1 - num.valid.days.log.v1)')
    if (grepl('total.num.steps.log.v2', var.)) fm. <- paste(fm., ' + offset(num.valid.days.log.v2) + I(total.num.steps.log.v1 - num.valid.days.log.v1) + I(total.num.steps.log.v3 - num.valid.days.log.v3)')
    if (!grepl('total.num.steps.log.v2', var.)) fm. <- paste(fm., ' + I(total.num.steps.log.v1 - num.valid.days.log.v1) + I(total.num.steps.log.v2 - num.valid.days.log.v2)')
    if (var. %in% c("hba1c.log.v2", "glu.log.v2", "insulin.log.v2")) fm. <- paste(c(fm., sub('v2', 'v1', var.)), collapse=' + ')
    fm.list <- c(fm.list, as.formula(fm.)); rm(fm.)
  }; rm(var.)
  for (var. in m8.list) {
    # var. <- m2.list[1]
    fm. <- paste(var., "~ (", paste(c(
      m0.list[!m0.list %in% c('total.num.steps.log.v1')], c('m4.compliance')
      , m4.list[!m4.list %in% c('total.num.steps.log.v2')]
      , 'm12.compliance'[!'m12.compliance' %in% var.]
    ), collapse=' + '), ')')
    fm. <- paste(fm., ' + I(total.num.steps.log.v1 - num.valid.days.log.v1) + I(total.num.steps.log.v2 - num.valid.days.log.v2)')
    fm.list <- c(fm.list, as.formula(fm.)); rm(fm.)
  }; rm(var.)
  for (var. in m12.list) {
    fm. <- paste(var., "~ (", paste(c(
      m0.list[!m0.list %in% c('total.num.steps.log.v1')]
      , c('m4.compliance', 'm12.compliance')
      , m12.list[!m12.list %in% c(var., 'total.num.steps.log.v3')]
    ), collapse=' + '), ')')
    fm. <- paste(fm., '')
    if (grepl('total.num.steps.log.v3', var.)) fm. <- paste(fm., ' + offset(num.valid.days.log.v3) + I(total.num.steps.log.v1 - num.valid.days.log.v1) + I(total.num.steps.log.v2 - num.valid.days.log.v2)')
    if (!grepl('total.num.steps.log.v3', var.)) fm. <- paste(fm., ' + I(total.num.steps.log.v1 - num.valid.days.log.v3) + I(total.num.steps.log.v1 - num.valid.days.log.v1)')
    if (var. %in% c("hba1c.log.v3", "glu.log.v3", "insulin.log.v3")) fm. <- paste(c(fm., sub('v3', 'v2', var.)), collapse=' + ')
    fm.list <- c(fm.list, as.formula(fm.)); rm(fm.)
  }; rm(var.)
  fm.list <- as.list(fm.list)
  names(fm.list) <- c(m0.list, m2.list, m4.list, m8.list, m12.list)
  fm.list$m4.compliance <- as.formula('m4.compliance ~ 1')
  fm.list$m12.compliance <- as.formula('m12.compliance ~ 1')
  rm(aux.list, m0.list, m2.list, m4.list, m8.list, m12.list)
}
```

```
## Imputation methods
{
  library(mice)
  mice.fit <- mice(
    data=imp.data
    , m=1, maxit=0
    , blocks=var.list
    , visitSequent=var.list
    , formulas=fm.list
  )
  # mice.fit$loggedEvents
  method. <- mice.fit$method
  method.[names(method.) == 'm4.compliance'] <- (
    "~ I((
        0.5*as.numeric(as.character(m0.5))*((ice.grp == 'Completer') + (ice.grp == 'Discontinue diet')*(drop.out.time >= 0.5))
        + 0.5*as.numeric(as.character(m1))*((ice.grp == 'Completer') + (ice.grp == 'Discontinue diet')*(drop.out.time >= 1))
        + 1*as.numeric(as.character(m2))*((ice.grp == 'Completer') + (ice.grp == 'Discontinue diet')*(drop.out.time >= 2))
        + 1*as.numeric(as.character(m3))*((ice.grp == 'Completer') + (ice.grp == 'Discontinue diet')*(drop.out.time >= 3))
        + 0.5*as.numeric(as.character(m3.5))*((ice.grp == 'Completer') + (ice.grp == 'Discontinue diet')*(drop.out.time >= 3.5))
        + 0.5*as.numeric(as.character(m4))*((ice.grp == 'Completer') + (ice.grp == 'Discontinue diet')*(drop.out.time >= 4))
      )/14/4)"
  )
  method.[names(method.) == 'm12.compliance'] <- (
    "~ I((
         4*as.numeric(as.character(m8))*((ice.grp == 'Completer') + (ice.grp == 'Discontinue diet')*(drop.out.time >= 8))
         + 4*as.numeric(as.character(m12))*((ice.grp == 'Completer') + (ice.grp == 'Discontinue diet')*(drop.out.time >= 12))
        )/14/8)"
  )
  method.

  rm(mice.fit)
}
ls()

## Imputation per treatment group
M. <- 100; N.iter <- 100
for (group. in 0:1) {

  library(mice)
  mice.fit <- mice(
    data=imp.data[imp.data$group == group.,]
    , m=M., maxit=N.iter
    , blocks=var.list
    , visitSequent=var.list
    , formulas=fm.list
    , method=method.
    , print=T
  )
  mice.fit$loggedEvents
  # plot(mice.fit)

  if (group. == 0) mice.fit.g0 <- mice.fit
  if (group. == 1) mice.fit.g1 <- mice.fit
  rm(mice.fit)

}; rm(group., M., N.iter)
rm(var.list, method., fm.list, imp.data)
ls()



####################
## Primary Analysis
####################

## HbA1c at 4 months
library(mice)
fm. <- "lm(exp(hba1c.log.v2) - exp(hba1c.log.v1) ~ group + sex + state + exp(hba1c.log.v1))"
output <- summary(pool(with(
  data = rbind(mice.fit.g0, mice.fit.g1)
  , expr = eval(parse(text=fm.))
))); output
grp.est <- output$estimate[2]
grp.est.se <- output$std.error[2]
grp.est.df <- output$df[2]
grp.est.p <- output$p.value[2]
if (grp.est.p < 0.001) grp.est.p <- '<0.001'
if (grp.est.p >= 0.01) grp.est.p <- format(round(grp.est.p, 2), nsmall=2)
if (grp.est.p < 0.01 & grp.est.p >= 0.001) grp.est.p <- format(round(grp.est.p, 3), nsmall=3)
paste(
  'iTRE - CP: '
  , format(round(grp.est, 2), nsmall=2), ' ['
  , format(round(grp.est - qt(1-0.05/2, df=grp.est.df)*grp.est.se, 2), nsmall=2), ', '
  , format(round(grp.est + qt(1-0.05/2, df=grp.est.df)*grp.est.se, 2), nsmall=2), ']; p='
  , grp.est.p, sep=''
); rm(grp.est, grp.est.se, grp.est.df, grp.est.p, output, fm.)
```

```
####################
## Secondary outcome example analyses
####################

## HbA1c at 12 months
library(mice)
fm. <- "lm(exp(hba1c.log.v3) - exp(hba1c.log.v1) ~ group + sex + state + exp(hba1c.log.v1))"
output <- summary(pool(with(
  data = rbind(mice.fit.g0, mice.fit.g1)
  , expr = eval(parse(text=fm.))
))); output
grp.est <- output$estimate[2]
grp.est.se <- output$std.error[2]
grp.est.df <- output$df[2]
paste(
  'iTRE - CP: '
  , format(round(grp.est, 2), nsmall=2), ' ['
  , format(round(grp.est - qt(1-0.05/2, df=grp.est.df)*grp.est.se, 2), nsmall=2), ', '
  , format(round(grp.est + qt(1-0.05/2, df=grp.est.df)*grp.est.se, 2), nsmall=2), ']'
  , sep=''
); rm(grp.est, grp.est.se, grp.est.df, output, fm.)


## Other Secondary outcomes will also adjust for their baseline and HbA1c at baseline
## Glucose at 4 months
library(mice)
fm. <- "lm(exp(glu.log.v2) - exp(glu.log.v1) ~ group + sex + state + exp(glu.log.v1) + exp(hba1c.log.v1))"
output <- summary(pool(with(
  data = rbind(mice.fit.g0, mice.fit.g1)
  , expr = eval(parse(text=fm.))
))); output
grp.est <- output$estimate[2]
grp.est.se <- output$std.error[2]
grp.est.df <- output$df[2]
paste(
  'iTRE - CP: '
  , format(round(grp.est, 2), nsmall=2), ' ['
  , format(round(grp.est - qt(1-0.05/2, df=grp.est.df)*grp.est.se, 2), nsmall=2), ', '
  , format(round(grp.est + qt(1-0.05/2, df=grp.est.df)*grp.est.se, 2), nsmall=2), ']'
  , sep=''
); rm(grp.est, grp.est.se, grp.est.df, output, fm.)

rm(mice.fit.g0, mice.fit.g1, MNAR.type)
ls()
########################## [illegible] ##############################
```

CGM code

```
CGM.baseline.fn <- function(y, t, threshold=1, smooth.range=4*2*3 + 1, iter=8) {
  # y = time series observations
  # t = time of observations
  # smooth.range = #obs/hr * both directions * duration (hr) + 1 (median)
  # threshold = deviations from local mean (Note that threshold can be defined as residual SD
  #, but not needed here.)
  # iter = number of iterations for

  ## Ensure y is correctly ordered
  y <- y[order(t)]

  ## Ensure no two consecutive readings with the same value the same
  cond. <- c(F, y[-1] == y[-length(y)])
  y[cond.] <- y[cond.] + 1/100/sum(cond.)
  rm(cond.)

  ## Generate baseline smooth y without large deviations (large defined as > threshold)
  smoothed.y <- y
  signals <- rep(0,length(y))
  for (it. in 1:iter) {
    # it. <- 1
    library(zoo)
    smoothed.y[signals != 0] <- NA
    smoothed.y <- rollapply(
      data=smoothed.y
      , width=smooth.range
      , partial=T
      , by.column = FALSE
      , FUN=function(x) { return(mean(x, na.rm=T))})
    smoothed.y <- na.fill(smoothed.y, "extend")

    ## When smoothed.y too high due to NA imputing reduce by threshold
    smoothed.y[y < smoothed.y - threshold] <- smoothed.y[y < smoothed.y - threshold] - threshold
    smoothed.y <- rollmean(smoothed.y, k=ceiling((smooth.range - 1)/2), fill='extend')

    # Asymmetric signal detection.
    signals <- rep(0,length(y))
    signals[y > smoothed.y + threshold*it./iter] <- 1

  }; rm(it.)

  ## Find peaks in signals, separated by dips
  neg.change <- c(y[-length(y)] < y[-1], F) & c(F, y[-1] < y[-length(y)])
  # ## exclude dips that consist of a single time point.
  pos.change <- c(y[-length(y)] > y[-1], F) & c(F, y[-1] > y[-length(y)])
  neg.change[neg.change & c(pos.change[-1], F) & c(F, pos.change[-length(y)])] <- F
  ## Set resulting dips to 0
  signals[neg.change & signals == 1] <- 0

  ## Peak = max of consecutive signals.
  signal.grp <- c(1, cumsum(signals[-length(y)] != signals[-1]) + 1)
  peaks <- y == tapply(y, signal.grp, max)[tapply(y, signal.grp,)] & signals == 1

  return(list("signals"=signals, "peaks"=peaks, "avgFilter"=smoothed.y))
  rm(y, t, threshold, iter, smooth.range, smoothed.y, neg.change, pos.change, signal.grp, signals)
}
```

ESM 4

```
#################
## MICE
#################

output <- NULL

## Number of simulated data-sets & iterations
M. <- 100; N.iter <- 100

## MI
set.seed(1234)
time. <- Sys.time()
par(mfrow=c(4,4))
for (MNAR.type in 0:1) {

  library(mice)
  # Set up variables
  {
    m0.list <- c(
      'state', 'age', 'sex', 'hba1c.screening.log'
      , "hba1c.log.v0", "glu.log.v0", "insulin.log.v0"
      , 'total.num.steps.log.v0', "cgm.night.mean.v0"
    )
    sum(!m0.list %in% names(imp.data)) # 0
    m0.list <- m0.list[order(colSums(is.na(imp.data[,m0.list])))]

    m2.list <- c('ice.grp', paste('m', c(0.5, 1, 1.5, 2, 2.5, 3, 3.5, 4),
sep=''), "m4.compliance")
    sum(!m2.list %in% names(imp.data)) # 0

    m4.list <- c(
      "hba1c.log.v2", "glu.log.v2", "insulin.log.v2"
      , "total.num.steps.log.v2", "cgm.night.mean.v2"
    )
    sum(!m4.list %in% names(imp.data)) # 0
    m4.list <- m4.list[order(colSums(is.na(imp.data[,m4.list])))]

    m8.list <- c(paste('m', c(6, 8, 10, 11.5, 12), sep=''),
"m12.compliance")
    sum(!m8.list %in% names(imp.data)) # 0

    m12.list <- c("hba1c.log.v5", "glu.log.v5", "insulin.log.v5",
"total.num.steps.log.v5")
    sum(!m12.list %in% names(imp.data)) # 0
    m12.list <- m12.list[order(colSums(is.na(imp.data[,m12.list])))]

    aux.list <- c("id", "group", "drop.out.time",
"num.valid.days.log.v0", "num.valid.days.log.v2",
"num.valid.days.log.v5")
    sum(!aux.list %in% names(imp.data)) # 0

    table(table(c(m0.list, m2.list, m4.list, m8.list, m12.list,
aux.list))) # all 1s

    var.list <- c(m0.list, m2.list, m4.list, m8.list, m12.list)
    temp.data <- imp.data[, c(var.list, aux.list)]
```

```
    colSums(is.na(temp.data))

  }

  ## MNAR: Do not impute ICE.grp, set at worst case: G1 discontinue vs G0 
completer
  if (MNAR.type == 1) {
    temp.data$ice.grp[temp.data$group. == 'B' & is.na(temp.data$ice.grp)] 
<- 'Completer'
    temp.data$ice.grp[temp.data$group. == 'A' & is.na(temp.data$ice.grp)] 
<- 'Discontinue diet'
  }

  # Set up functions
  {
    fm.list <- NULL
    for (var. in m0.list) {
      # var. <- m0.list[4]
      fm. <- paste(var., "~", paste(m0.list[!m0.list %in% c(var., 
'total.num.steps.log.v0')], collapse=' + '))
      if (!grepl('total.num.steps.log', var.)) fm. <- paste(fm., ' + 
I(total.num.steps.log.v0 - num.valid.days.log.v0)')
      if (grepl('total.num.steps.log', var.)) fm. <- paste(fm., ' + 
offset(num.valid.days.log.v0)')
      if (var. %in% c("hba1c.log.v0", "glu.log.v0", "insulin.log.v0")) {
        fm. <- paste(c(fm., sub('v0', 'v2', var.), sub('v0', 'v5', 
var.)), collapse=' + ')
      }
      if (var. %in% c("cgm.night.mean.v0")) {
        fm. <- paste(c(fm., sub('v0', 'v2', var.)), collapse=' + ')
      }
      fm.list <- c(fm.list, as.formula(fm.)); rm(fm.)
    }; rm(var.)
    for (var. in m2.list) {
      # var. <- m2.list[1]
      fm. <- paste(var., "~ (", paste(c(
        m0.list[!m0.list %in% c('total.num.steps.log.v0')]
        , c('ice.grp', 'm4.compliance')[!c('ice.grp', 'm4.compliance') 
%in% var.]
        , m4.list[!m4.list %in% c('total.num.steps.log.v2')]
      ), collapse=' + '), ')')
      fm. <- paste(fm., ' + I(total.num.steps.log.v0 - 
num.valid.days.log.v0) + I(total.num.steps.log.v2 - 
num.valid.days.log.v2)')
      fm.list <- c(fm.list, as.formula(fm.)); rm(fm.)
    }; rm(var.)
    for (var. in m4.list) {
      fm. <- paste(var., "~ (", paste(c(
        m0.list[!m0.list %in% c('total.num.steps.log.v0')]
        , c('m4.compliance')
        , m4.list[!m4.list %in% c(var., 'total.num.steps.log.v2')]
      ), collapse=' + '), ')')
      fm. <- paste(fm., ' + I(total.num.steps.log.v0 - 
num.valid.days.log.v0)')
      if (grepl('total.num.steps.log.v2', var.)) fm. <- paste(fm., ' + 
offset(num.valid.days.log.v2) + I(total.num.steps.log.v0 - 
num.valid.days.log.v0)')
```

```
      if (grepl('total.num.steps.log.v2', var.)) fm. <- sub(' [+]
ice.grp', '', fm.)
      if (!grepl('total.num.steps.log.v2', var.)) fm. <- paste(fm., ' +
I(total.num.steps.log.v0 - num.valid.days.log.v0) +
I(total.num.steps.log.v2 - num.valid.days.log.v2)')
      if (var. %in% c("hba1c.log.v2", "glu.log.v2", "insulin.log.v2")) {
        fm. <- paste(c(fm., sub('v2', 'v5', var.)), collapse=' + ')
      }
      fm.list <- c(fm.list, as.formula(fm.)); rm(fm.)
    }; rm(var.)
    for (var. in m8.list) {
      # var. <- m8.list[1]
      fm. <- paste(var., "~ (", paste(c(
        m0.list[!m0.list %in% c('total.num.steps.log.v0')]
        , c('ice.grp', 'm4.compliance')
        , m4.list[!m4.list %in% c('total.num.steps.log.v0')]
        , "m12.compliance"[!"m12.compliance" %in% var.]
      ), collapse=' + '), ')')
      fm. <- paste(fm., ' + I(total.num.steps.log.v0 -
num.valid.days.log.v0) + I(total.num.steps.log.v2 -
num.valid.days.log.v2)')
      fm.list <- c(fm.list, as.formula(fm.)); rm(fm.)
    }; rm(var.)
    for (var. in m12.list) {
      # var. <- m12.list[4]
      fm. <- paste(var., "~ (", paste(c(
        m0.list[!m0.list %in% c('total.num.steps.log.v0')]
        , c('m4.compliance', 'm12.compliance')
        , m12.list[!m12.list %in% c(var., 'total.num.steps.log.v5')]
      ), collapse=' + '), ')')
      fm. <- paste(fm., '')
      if (grepl('total.num.steps.log.v5', var.)) fm. <- paste(fm., ' +
offset(num.valid.days.log.v5) + I(total.num.steps.log.v0 -
num.valid.days.log.v0) + I(total.num.steps.log.v2 -
num.valid.days.log.v2)')
      if (grepl('total.num.steps.log.v5', var.)) fm. <- sub(' [+]
ice.grp', '', fm.)
      if (!grepl('total.num.steps.log.v5', var.)) fm. <- paste(fm., ' +
I(total.num.steps.log.v5 - num.valid.days.log.v5) +
I(total.num.steps.log.v0 - num.valid.days.log.v0)')
      if (var. %in% c("hba1c.log.v5", "glu.log.v5", "insulin.log.v5")) {
        fm. <- paste(c(fm., sub('v5', 'v2', var.)), collapse=' + ')
      }
      fm.list <- c(fm.list, as.formula(fm.)); rm(fm.)
    }; rm(var.)
    fm.list <- as.list(fm.list)
    names(fm.list) <- c(m0.list, m2.list, m4.list, m8.list, m12.list)
    rm(m0.list, m2.list, m4.list, m8.list, m12.list)

    fm.list$m4.compliance <- as.formula('m4.compliance ~ 1')
    fm.list$m12.compliance <- as.formula('m12.compliance ~ 1')

    fm.list

  }

  library(mice)
  # Set up imputation methods
```

```
  {
    mice.fit <- mice(
      data=temp.data
      , m=1, maxit=0
      , blocks=var.list
      , visitSequent=var.list
      , formulas=fm.list
    )
    #mice.fit$loggedEvents
    method. <- mice.fit$method
    {
      method.[names(method.) == 'm4.compliance'] <- gsub('\n', '',
                                                          "~ I((
        0.5*as.numeric(as.character(m0.5))*((ice.grp == 'Completer') +
(ice.grp == 'Discontinue diet')*(drop.out.time >= 0.5))
        + 0.5*as.numeric(as.character(m1))*((ice.grp == 'Completer') +
(ice.grp == 'Discontinue diet')*(drop.out.time >= 1))
        + 0.5*as.numeric(as.character(m1.5))*((ice.grp == 'Completer') +
(ice.grp == 'Discontinue diet')*(drop.out.time >= 1.5))
        + 0.5*as.numeric(as.character(m2))*((ice.grp == 'Completer') +
(ice.grp == 'Discontinue diet')*(drop.out.time >= 2))
        + 0.5*as.numeric(as.character(m2.5))*((ice.grp == 'Completer') +
(ice.grp == 'Discontinue diet')*(drop.out.time >= 2.5))
        + 0.5*as.numeric(as.character(m3))*((ice.grp == 'Completer') +
(ice.grp == 'Discontinue diet')*(drop.out.time >= 3))
        + 0.5*as.numeric(as.character(m3.5))*((ice.grp == 'Completer') +
(ice.grp == 'Discontinue diet')*(drop.out.time >= 3.5))
        + 0.5*as.numeric(as.character(m4))*((ice.grp == 'Completer') +
(ice.grp == 'Discontinue diet')*(drop.out.time >= 4))
      )/14/4)"
      )
      method.[names(method.) == 'm12.compliance'] <- gsub('\n', '',
                                                           "~ I((
          2*as.numeric(as.character(m6))*((ice.grp == 'Completer') +
(ice.grp == 'Discontinue diet')*(drop.out.time >= 6))
          + 2*as.numeric(as.character(m8))*((ice.grp == 'Completer') +
(ice.grp == 'Discontinue diet')*(drop.out.time >= 8))
          + 2*as.numeric(as.character(m10))*((ice.grp == 'Completer') +
(ice.grp == 'Discontinue diet')*(drop.out.time >= 10))
          + 1.5*as.numeric(as.character(m11.5))*((ice.grp == 'Completer')
+ (ice.grp == 'Discontinue diet')*(drop.out.time >= 11.5))
          + 0.5*as.numeric(as.character(m12))*((ice.grp == 'Completer') +
(ice.grp == 'Discontinue diet')*(drop.out.time >= 12))
        )/14/8)"
      )
    }

  }

  options(warn=0)
  #time. <- Sys.time()
  for (group. in c('A', 'B')) {
    var.max <- length(var.list)
    mice.fit2 <- mice(
      data=temp.data[temp.data$group == group.,]
      , m=M., maxit=N.iter
      , blocks=var.list[1:var.max]
      , visitSequent=var.list[1:var.max]
```

```
      , formulas=fm.list[1:var.max]
      , method=method.[1:var.max]
      , print=F
    ); rm(var.max)
    mice.fit2$loggedEvents

    plot(mice.fit2)

    if (group. == 'A') mice.fit.g0 <- mice.fit2
    if (group. == "B") mice.fit.g1 <- mice.fit2

  }; rm(group.)
  rm(aux.list, var.list)
  rm(mice.fit, mice.fit2, method., fm.list)

  mice.full.fit <- rbind(mice.fit.g0, mice.fit.g1); rm(mice.fit.g0,
mice.fit.g1)

  ## Store solutions
  library(mice)
  var.list <- c('hba1c.log', 'insulin.log', 'glu.log',
'total.num.steps.log', 'cgm.night.mean')
  for (var. in var.list) {
    # var. <- var.list[1]
    for (visit. in c('.v2', '.v5')) {
      # visit. <- '.v2'
      if (!(var. == 'cgm.night.mean' & visit. == '.v5')) {
        output <- rbind(output, c(
          c('MI', 'MI-worst')[0:1 == MNAR.type]
          , 'diff - orig'
          , sub('.log', '', var.)
          , toupper(substring(visit.,2,3))
          , sum(rowSums(is.na(complete(mice.full.fit, m=1)[[1]])) == 0)
          , summary(pool(with(
            data = mice.full.fit
            , expr = eval(parse(text=paste('lm(exp(', var., visit., ') -
exp(', var., '.v0) ~ 0 + group + sex + state + I(exp(', var., '.v0) -
mean(exp(', var., '.v0))))', sep='')))
          )))$estimate[1]
          , summary(pool(with(
            data = mice.full.fit
            , expr = eval(parse(text=paste('lm(exp(', var., visit., ') -
exp(', var., '.v0) ~ 0 + group + sex + state + I(exp(', var., '.v0) -
mean(exp(', var., '.v0))))', sep='')))
          )))$std.error[1]
          , summary(pool(with(
            data = mice.full.fit
            , expr = eval(parse(text=paste('lm(exp(', var., visit., ') -
exp(', var., '.v0) ~ 0 + group + sex + state + I(exp(', var., '.v0) -
mean(exp(', var., '.v0))))', sep='')))
          )))$estimate[2]
          , summary(pool(with(
            data = mice.full.fit
            , expr = eval(parse(text=paste('lm(exp(', var., visit., ') -
exp(', var., '.v0) ~ 0 + group + sex + state + I(exp(', var., '.v0) -
mean(exp(', var., '.v0))))', sep='')))
          )))$std.error[2]
          , summary(pool(with(
```

```
            data = mice.full.fit
            , expr = eval(parse(text=paste('lm(exp(', var., visit., ') -
exp(', var., '.v0) ~ group + sex + state + I(exp(', var., '.v0) -
mean(exp(', var., '.v0))))', sep='')))
          )))$estimate[2]
          , summary(pool(with(
            data = mice.full.fit
            , expr = eval(parse(text=paste('lm(exp(', var., visit., ') -
exp(', var., '.v0) ~ group + sex + state + I(exp(', var., '.v0) -
mean(exp(', var., '.v0))))', sep='')))
          )))$std.error[2]
        ))
        if (MNAR.type == 1) output <- rbind(output, c(
          c('CC')
          , 'diff - orig'
          , sub('.log', '', var.)
          , toupper(substring(visit.,2,3))
          , sum(rowSums(is.na(imp.data[,c(paste(var., visit., sep=''),
paste(var., '.v0', sep=''), 'group', 'sex', 'state')])) == 0)
          , summary(eval(parse(text=paste('lm(exp(', var., visit., ') -
exp(', var., '.v0) ~ 0 + group + sex + state + I(exp(', var., '.v0) -
mean(exp(', var., '.v0), na.rm=T)), data=imp.data)',
sep=''))))$coef[1,1:2]
          , summary(eval(parse(text=paste('lm(exp(', var., visit., ') -
exp(', var., '.v0) ~ 0 + group + sex + state + I(exp(', var., '.v0) -
mean(exp(', var., '.v0), na.rm=T)), data=imp.data)',
sep=''))))$coef[2,1:2]
          , summary(eval(parse(text=paste('lm(exp(', var., visit., ') -
exp(', var., '.v0) ~ group + sex + state + I(exp(', var., '.v0) -
mean(exp(', var., '.v0), na.rm=T)), data=imp.data)',
sep=''))))$coef[2,1:2]
        ))
      }
    }; rm(visit.)
  }; rm(var., var.list)
  for (var. in 'total.num.steps.log') {
    for (visit. in c('.v2', '.v5')) {
      {
        output <- rbind(output, c(
          c('MI', 'MI-worst')[0:1 == MNAR.type]
          , 'diff - orig'
          , 'mean.daily.steps'
          , toupper(substring(visit.,2,3))
          , sum(rowSums(is.na(complete(mice.full.fit, m=1)[[1]])) == 0)
          , summary(pool(with(
            data = mice.full.fit
            , expr = eval(parse(text=paste('lm(exp(', var., visit., ' -
num.valid.days.log', visit., ') - exp(', var., '.v0 -
num.valid.days.log.v0) ~ 0 + group + sex + state + I(exp(', var., '.v0 -
num.valid.days.log.v0) - mean(exp(', var., '.v0 -
num.valid.days.log.v0))))', sep='')))
          )))$estimate[1]
          , summary(pool(with(
            data = mice.full.fit
            , expr = eval(parse(text=paste('lm(exp(', var., visit., ' -
num.valid.days.log', visit., ') - exp(', var., '.v0 -
num.valid.days.log.v0) ~ 0 + group + sex + state + I(exp(', var., '.v0 -
```

```
num.valid.days.log.v0) - mean(exp(', var., '.v0 -
num.valid.days.log.v0))))', sep='')))
          )))$std.error[1]
          , summary(pool(with(
            data = mice.full.fit
            , expr = eval(parse(text=paste('lm(exp(', var., visit., ' -
num.valid.days.log', visit., ') - exp(', var., '.v0 -
num.valid.days.log.v0) ~ 0 + group + sex + state + I(exp(', var., '.v0 -
num.valid.days.log.v0) - mean(exp(', var., '.v0 -
num.valid.days.log.v0))))', sep='')))
          )))$estimate[2]
          , summary(pool(with(
            data = mice.full.fit
            , expr = eval(parse(text=paste('lm(exp(', var., visit., ' -
num.valid.days.log', visit., ') - exp(', var., '.v0 -
num.valid.days.log.v0) ~ 0 + group + sex + state + I(exp(', var., '.v0 -
num.valid.days.log.v0) - mean(exp(', var., '.v0 -
num.valid.days.log.v0))))', sep='')))
          )))$std.error[2]
          , summary(pool(with(
            data = mice.full.fit
            , expr = eval(parse(text=paste('lm(exp(', var., visit., ' -
num.valid.days.log', visit., ') - exp(', var., '.v0 -
num.valid.days.log.v0) ~ group + sex + state + I(exp(', var., '.v0 -
num.valid.days.log.v0) - mean(exp(', var., '.v0 -
num.valid.days.log.v0))))', sep='')))
          )))$estimate[2]
          , summary(pool(with(
            data = mice.full.fit
            , expr = eval(parse(text=paste('lm(exp(', var., visit., ' -
num.valid.days.log', visit., ') - exp(', var., '.v0 -
num.valid.days.log.v0) ~ group + sex + state + I(exp(', var., '.v0 -
num.valid.days.log.v0) - mean(exp(', var., '.v0 -
num.valid.days.log.v0))))', sep='')))
          )))$std.error[2]
        ))
        if (MNAR.type == 1) output <- rbind(output, c(
          c('CC')
          , 'diff - orig'
          , 'mean.daily.steps'
          , toupper(substring(visit.,2,3))
          , sum(rowSums(is.na(imp.data[,c(paste(var., visit., sep=''),
paste(var., '.v0', sep=''), 'group', 'sex', 'state')])) == 0)
          , summary(eval(parse(text=paste('lm(exp(', var., visit., ' -
num.valid.days.log', visit., ') - exp(', var., '.v0 -
num.valid.days.log.v0) ~ 0 + group + sex + state + I(exp(', var., '.v0 -
num.valid.days.log.v0) - mean(exp(', var., '.v0 - num.valid.days.log.v0),
na.rm=T)), data=imp.data)', sep=''))))$coef[1,1:2]
          , summary(eval(parse(text=paste('lm(exp(', var., visit., ' -
num.valid.days.log', visit., ') - exp(', var., '.v0 -
num.valid.days.log.v0) ~ 0 + group + sex + state + I(exp(', var., '.v0 -
num.valid.days.log.v0) - mean(exp(', var., '.v0 - num.valid.days.log.v0),
na.rm=T)), data=imp.data)', sep=''))))$coef[2,1:2]
          , summary(eval(parse(text=paste('lm(exp(', var., visit., ' -
num.valid.days.log', visit., ') - exp(', var., '.v0 -
num.valid.days.log.v0) ~ group + sex + state + I(exp(', var., '.v0 -
num.valid.days.log.v0) - mean(exp(', var., '.v0 - num.valid.days.log.v0),
na.rm=T)), data=imp.data)', sep=''))))$coef[2,1:2]
```

```
        ))
      }
    }; rm(visit.)
  }; rm(var.)
  rm(temp.data, mice.full.fit)

  output

}; rm(MNAR.type, M., N.iter)
par(mfrow=c(1,1))
Sys.time() - time.; rm(time.)
output <- as.data.frame(output)
names(output) <- c('analysis', 'type', 'var', 'visit', 'n', 'est.A',
'se.A', 'est.B', 'se.B', 'est.diff', 'se.diff')
for (col. in 5:ncol(output)) output[,col.] <- as.numeric(output[,col.]);
rm(col.)

# HbA1c (%) = 0.09148 √ó HbA1c (mmol/mol) + 2.152
# Delta HbA1c (%) = 0.09148 √ó Delta HbA1c (mmol/mol)

## Include Hba1c on % scale
temp.out <- output[output$var == 'hba1c',]
temp.out$var <- 'hba1c_perc'
for (var. in c('est.A', 'se.A', 'est.B', 'se.B', 'est.diff', 'se.diff'))
{
  temp.out[,var.] <- (temp.out[,var.]) * 0.09148
}; rm(var.)
output <- rbind(output, temp.out); rm(temp.out)

source(paste(project.loc, 'Progs - Revisions Jan 2026/Z Table functions
20170127.r', sep=''))
output$ci.A <- ci.pretty.fn(output$est.A, output$se.A)
output$p.A <- p.value.fn(2*pnorm(-abs(output$est.A/output$se.A)))
output$ci.B <- ci.pretty.fn(output$est.B, output$se.B)
output$p.B <- p.value.fn(2*pnorm(-abs(output$est.B/output$se.B)))
output$ci.diff <- ci.pretty.fn(output$est.diff, output$se.diff)
output$p.diff <- p.value.fn(2*pnorm(-
abs(output$est.diff/output$se.diff)))
rm(list=ls(pattern='.fn'))



######################### END ############################
######################### END ############################
######################### END ############################
######################### END ############################
```

```
CGM.baseline.fn <- function(y, t, threshold=1, smooth.range=4*2*3 + 1, iter=8) {
  # y = time series observations
  # t = time of observations
  # smooth.range = #obs/hr * both directions * duration (hr) + 1 (median)
  # threshold = deviations from local mean (Note that threshold can be defined as residual SD
  #, but not needed here.)
  # iter = number of iterations for

  ## Ensure y is correctly ordered
  y <- y[order(t)]

  ## Ensure no two consecutive readings with the same value the same
  cond. <- c(F, y[-1] == y[-length(y)])
  y[cond.] <- y[cond.] + 1/100/sum(cond.)
  rm(cond.)

  ## Generate baseline smooth y without large deviations (large defined as > threshold)
  smoothed.y <- y
  signals <- rep(0,length(y))
  for (it. in 1:iter) {
    # it. <- 1
    library(zoo)
    smoothed.y[signals != 0] <- NA
    smoothed.y <- rollapply(
      data=smoothed.y
      , width=smooth.range
      , partial=T
      , by.column = FALSE
      , FUN=function(x) { return(mean(x, na.rm=T))})
    smoothed.y <- na.fill(smoothed.y, "extend")

    ## When smoothed.y too high due to NA imputing reduce by threshold
    smoothed.y[y < smoothed.y - threshold] <- smoothed.y[y < smoothed.y - threshold] - threshold
    smoothed.y <- rollmean(smoothed.y, k=ceiling((smooth.range - 1)/2), fill='extend')

    # Asymmetric signal detection.
    signals <- rep(0,length(y))
    signals[y > smoothed.y + threshold*it./iter] <- 1

  }; rm(it.)

  ## Find peaks in signals, separated by dips
  neg.change <- c(y[-length(y)] < y[-1], F) & c(F, y[-1] < y[-length(y)])
  # ## exclude dips that consist of a single time point.
  pos.change <- c(y[-length(y)] > y[-1], F) & c(F, y[-1] > y[-length(y)])
  neg.change[neg.change & c(pos.change[-1], F) & c(F, pos.change[-length(y)])] <- F
  ## Set resulting dips to 0
  signals[neg.change & signals == 1] <- 0

  ## Peak = max of consecutive signals.
  signal.grp <- c(1, cumsum(signals[-length(y)] != signals[-1]) + 1)
  peaks <- y == tapply(y, signal.grp, max)[tapply(y, signal.grp,)] & signals == 1

  return(list("signals"=signals, "peaks"=peaks, "avgFilter"=smoothed.y))
  rm(y, t, threshold, iter, smooth.range, smoothed.y, neg.change, pos.change, signal.grp, signals)
}
```
